# Barriers and enablers for older adults participating in a home-based pragmatic exercise program delivered and monitored by Amazon Alexa: a qualitative study

**DOI:** 10.1186/s12877-022-02963-2

**Published:** 2022-03-25

**Authors:** Paul Jansons, Jackson Fyfe, Jack Dalla Via, Robin M. Daly, Eugene Gvozdenko, David Scott

**Affiliations:** 1 Institute for Physical Activity and Nutrition (IPAN), School of Exercise and Nutrition Sciences, Deakin University, Geelong, Australia; 2 Department of Medicine, School of Clinical Sciences at Monash Health, Monash University, Clayton, VIC Australia; 3 Institute for Nutrition Research, School of Medical and Health Sciences, Edith Cowan University, Perth, WA Australia; 4 Great Australian Pty. Ltd, Keysborough, VIC Australia

## Abstract

**Background:**

The remote delivery and monitoring of individually-tailored exercise programs using voice-controlled intelligent personal assistants (VIPAs) that support conversation-based interactions may be an acceptable alternative model of digital health delivery for older adults. The aim of this study was to evaluate the enablers and barriers for older adults participating in a home-based exercise program delivered and monitored by VIPAs.

**Method:**

This qualitative study used videoconferencing to conduct semi-structured interviews following a 12-week, prospective single-arm pilot study in 15 adults aged 60 to 89 years living alone in the community. All participants were prescribed an individualized, brief (10 min, 2–4 times per day), home-based muscle strengthening and balance exercise program delivered and monitored using an Amazon Echo Show 5 device (Alexa). Qualitative interview data were analysed using inductive thematic analysis.

**Results:**

All 15 participants (aged 70.3 ± 4.3 years, mean ± SD) attended the semi-structured interview. Themes including enjoyability and ease of use, social engagement and motivation were enablers for participation in the exercise program. Errors in voice recognition, lack of feedback, and preference for other existing digital health modes of exercise delivery were barriers associated with the Alexa technology.

**Conclusions:**

This qualitative study identified enablers and barriers associated with using an Alexa device to deliver and monitor an individualized, home-based exercise program in older adults living alone. Future interventions using VIPAs should focus on reducing technical errors, providing regular exercise feedback, and comparing participants’ experiences of exercise programs delivered by VIPAs to programs delivered via other digital health tools.

**Supplementary Information:**

The online version contains supplementary material available at 10.1186/s12877-022-02963-2.

## Introduction

Emerging evidence suggests digital health technologies are safe and effective models of service delivery for individualised exercise prescription to manage various chronic diseases [1], but interventions supported by these technologies can be challenging for older adults. [2]. At present, older adults participating in interventions using common asynchronous digital health tools (e.g., telephone monitoring) report a lack of feedback and needing greater self-motivation to complete their exercises at home [3]. Videoconferencing support can enable instant feedback and reassurance (e.g., regarding correct exercise technique) to the older adult [2]. However, access to an adequate device that supports videoconferencing [4] and difficultly to set aside time to exercise at scheduled appointments were reported barriers for older adults with these technologies [5]. Smartphone or web applications may reduce the time burden for older adults by allowing greater flexibility to participate in an exercise program at home. However, these tools can require significant training, meaning they may have low feasibility as a digital health tool for older adults [6].

Digital health platforms supporting conversation-based interactions may enhance adherence to remotely-delivered exercise interventions in older adults, because the human-like attributes associated with these technologies may elicit a sense of familiarity, social presence, and human engagement [7]. To this end, we developed software that allows health professionals to remotely deliver individualized exercise programs using voice-controlled intelligent personal assistants (VIPAs), specifically, Amazon Alexa Echo Show devices. These devices display audio and video content, and can interpret human speech, therefore reducing technology burden for patients (i.e., avoiding the need for use of digital inputs to interact with the program). Importantly, our software allows for convenient asynchronous interaction between patients and clinicians that may be more acceptable to older adults by providing greater flexibility, convenience, and easier integration into activities of daily living.

A qualitative review of 73,549 verified Amazon.com purchase reviews of Amazon Alexa Echo devices (which only have audio, not video, capabilities) found older adults and caregivers reported positive themes of companionship and assistance in maintaining a level of independence for the older adults [8]. Caregivers reported that Alexa successfully facilitated several of their daily tasks, such as medication and appointment reminders, turning lights on and off using smart home technology via the Alexa, and adding items to shopping lists [8]. A remotely-delivered, home-based intervention that included general information, reminders and entertainment for 30 older adults delivered by Alexa Echo Show reported themes of human engagement and companionship for those who live alone [9].

Recently, we conducted the first individually-tailored, home-based exercise intervention delivered using Amazon Alexa Echo Show devices in older adults [10]. Our results demonstrated feasibility and safety of the intervention, as evidenced by the low attrition, high exercise adherence, no reported intervention-related adverse events, and an above-average self-reported system usability score for the Alexa program [10]. The aim of this nested qualitative study was to explore the enablers and barriers of community based older adults aged 60 to 89 years participating in the home-based exercise program delivered by Alexa.

## Methods

### Design

This qualitative study was nested within a 12-week community-based, prospective, single-arm pilot study in which adults aged 60 to 89 years were prescribed a home-based exercise program by an accredited exercise physiologist that was delivered remotely by Alexa (Deakin University Human Research Ethics Committee approval: 2020–166). The main findings from this trial have been previously reported [10]. The intervention and all data collection, including qualitative interviews via telephone calls, were conducted remotely in participants’ homes. All participants provided consent to participate in a qualitative interview after completion of the 12-week intervention. The study was reported in accordance with the consolidated criteria for reporting qualitative research [11].

### Participants

Potential participants were purposively sampled from the standalone intervention (ie, a single clinician prescribed the home-based exercise program independently) [10] via email invitation from a database of previous research participants who provided consent to be re-contacted for future trials. Participants were eligible to participate in the study if they were aged 60 to 89 years, living alone, English speaking, had access to a home Wi-Fi network, and did not have any medical condition that would pose difficulty when interacting with the Alexa device or performing unsupervised exercise safely including progressive neurological disorders, severe knee or hip osteoarthritis or recent fractures. Written informed consent was obtained from each participant directly. To reduce recall bias, participants meeting the inclusion criteria were contacted within 2 weeks of completing the trial to schedule an interview time. A detailed description of the elements specific to the participants eligibility and recruitment has been reported previously [10].

### Intervention

All participants completed a 12-week, individualized, home-based exercise program which was prescribed by a single clinician (accredited exercise physiologist with training in health coaching) and delivered via Alexa. Each participant was provided with an Amazon Alexa Echo Show 5 device featuring a 5.5-inch screen, a built-in camera for videoconferencing and a microphone array and speaker, which allowed the delivery of video and audio content and receipt of voice-controlled inputs by the user. We have developed an innovative software program (“Buddy Link”) that allows health professionals to remotely deliver personalized audio and video content to clients via Alexa devices.

Appropriate exercises were selected by the accredited exercise physiologist using the clinician interface in Buddy Link and the exercise program was delivered to participants via an Alexa using video demonstrations, accompanied with instructions in the form of audio and text on the display. All interactions between the exercise physiologist and the participants were conducted remotely (i.e. via Alexa device instructions or telephone/videoconference) and there was no pre-established relationship prior to enrolment in this study. Participants were prescribed an exercise program involving brief (10-min) but frequent (up to four times per day) sessions (consistent with an ‘exercise snacking’ approach) and incorporating simple exercises requiring minimal-to-no equipment. Specifically, ‘exercise snacking’ sessions were completed twice per day for the first four weeks of the intervention, three times per day for the second four weeks, and four times per day for the final four weeks. Each ‘exercise snacking’ session was 10 min in duration and involved four functional strength, weight-bearing impact and balance training exercises for the upper and lower body. Appropriate exercises were selected by the accredited exercise physiologist based on the principles of the *Osteo-cise: Strong Bones for Life* program [12]. Participants were instructed to perform exercise at a moderate intensity of approximately 4–6 on the 10-point modified Rating of Perceived Exertion (RPE) scale. Participants received a reminder on the Alexa device when a new program was available, and would access this content by saying the phrase, *“Alexa, open my Training Buddy”.* Participants were presented an audiovisual demonstration and instructions of each exercise. Participants would then be encouraged to complete the exercise in real-time alongside a visual demonstration provided by the instructor. Following each individual exercise and session, the Alexa device would broadcast questions to the participant to determine whether they completed the exercise, their levels of exertion during the exercise, and whether any potential adverse events/incidents occurred. For example: “*have you completed your exercise session, yes or no?*”, “*how hard did you find this exercise out of 10, 1 being very easy and 10 being extremely hard?*”, “*did you experience any pain or discomfort when completing this exercise, yes or no?*”. Participants’ voice responses to these questions were recorded and saved to the Buddy Link database, enabling the accredited exercise physiologist to review the responses weekly to tailor the program accordingly and modify or progress each participants exercise program as required. For example: if the participant self-reported their exertion level during a wall push 1 out of 10, being very easy, the exercise was progressed by increasing the range of movement to a push up off the kitchen bench. A detailed description of the elements specific to the intervention has been reported previously [10].

### Data collection

Semi-structured interviews were conducted via videoconferencing (using the Alexa device videoconferencing feature) by a study investigator (PJ) with experience in qualitative research. A set list of questions was designed to elicit responses to identify enablers and barriers of participating in the exercise intervention (Supplementary 1 Questions file). The study investigator asked further questions where necessary to clarify or obtain further information based on participant responses. All interviews conducted via videoconferencing were digitally voice recorded and were transcribed verbatim by a transcribing company (www.transcribeme.com. TranscribeMe Inc.). All qualitative data and associated transcripts were stored in a password-protected master database file. Only study investigators had access to these data and will be stored for a period of 15 years, after which it will be securely destroyed.

### Data analysis

A modified thematic framework was used to analyse the data [13]. NVivo computer software (version 12, QSR International Pty Ltd, Doncaster, Victoria, Australia) was used to code, chart and map the data. An iterative process was then used to test and retest the thematic framework. Two authors (PJ) and (JF) compared content and themes. Any disagreement was resolved by consensus moderation. The research team (JF, PJ, DS, JD, RD, EV) discussed and refined the final themes in the context of the research question. The number of participants interviewed was based on data sufficiency (judged by reviewing transcripts after each interview and discussion between the researchers). The participants did not have an opportunity to provide feedback on the findings.

## Results

All interviews were approximately 30 min in duration. Baseline participant demographics is presented in Table 1. Participants were aged 65 to 79 years (mean ± SD age 70.3 ± 4.3 years). Sixty per cent (9/15) of the participants were women, 53% (8/15) reported that both their parents were born in Australia and the same proportion (53%) had been educated at university level or similar, while two-thirds (10/15) were retired. Fifty-three percent (8/15) of participants reported having hypercholesteraemia, 47% (7/15) hypertension, and 40% (6/15) diabetes and osteoarthritis. All participants reported the presence of at least one chronic disease. No participant declined to participate in the semi-structured interviews.

**Table 1:** Baseline demographics

N	Intervention
	15
Age – Mean (SD)	70.3 (4.3)
Gender (Female) – n (%)	9 (60%)
Marital status – n (%)	
Widowed	3 (20.0%)
Divorced or separated	8 (53.3%)
Single	4 (26.7%)
Parents country of birth – n (%)	
Australia	8 (53.3%)
Other	7 (46.7%)
Highest level of education – n (%)	
Secondary/high school	3 (20%)
Technical or further educational institution	4 (26.7%)
University or other higher educational institution	8 (53.3%)
Current employment status – n (%)	
Retired	10 (66.7%)
Pension (including disability or sole pension)	3 (20%)
Medical conditions – n (%)	
Coronary heart disease	2 (13.3%)
Hypertension	7 (46.7%)
Hypercholesterolaemia	8 (53.3%)
Diabetes	6 (40%)
Asthma	2 (13.3%)
Any form of cancer	3 (20%)
Osteoarthritis	6 (40%)
Reported a least one chronic condition	15 (100%)

**Fig. 1:**
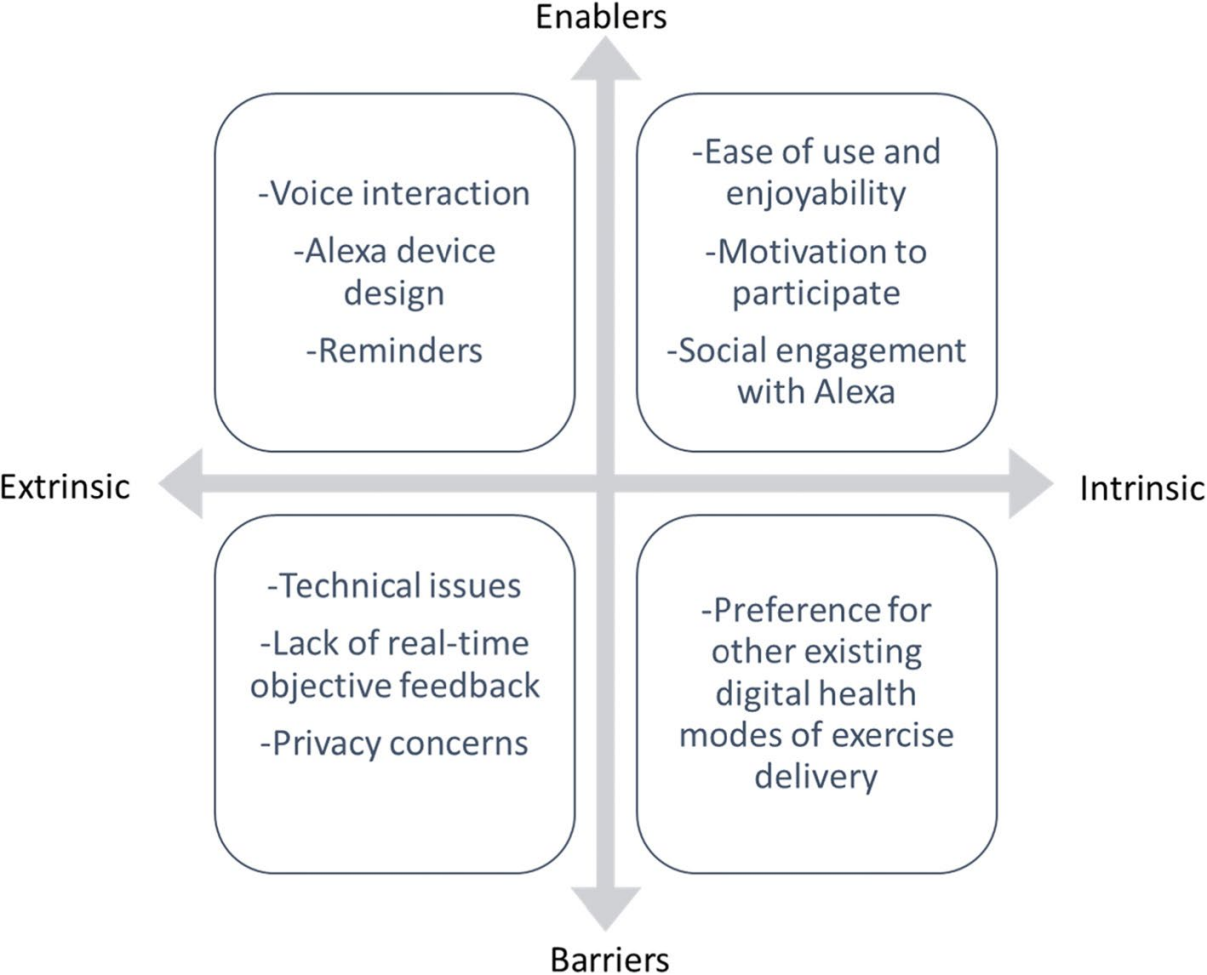
Enablers and barriers of using Alexa for the delivery of a home-based pragmatic exercise program

In summary, ten key themes were identified from the analysis of the semi-structured interviews and these are depicted in Fig. 1 which presents intrinsic and extrinsic enablers and barriers to using Alexa for the delivery of a home-based pragmatic exercise program. Themes including enjoyability and ease of use, social engagement, motivation, voice interaction, Alexa device design and reminding functionality were enablers for participation in the exercise program. Technical errors in voice recognition, lack of feedback, privacy concerns and preference for other existing digital health modes of exercise delivery were barriers associated with the Alexa technology.

### Theme 1: enablers of interacting with the Alexa to participate in the exercise intervention

Generally, participants reported several enablers to a home-based exercise program delivered and monitored by an Alexa supporting conversation-based interactions.

#### Voice interaction

Participants reported that using voice interactions to engage in the exercise program was user friendly.

*“I particularly like the voice thing if you can just say it without having to go and sort of manually touch the screen, yeah. That’s good.” (Participant 14, male, age 74).*

*“Yeah no, it’s good. You don’t have to touch it.” (Participant 9, female, age 65).*

*“A phone would probably do something similar…, but I mean it’s still you have to then go and dive into the app and all that sort of stuff, I guess.” (Participant 14, male, age 74).*

*“Oh yes, I didn’t need to be close to it to do the exercises. I could do it from a distance and it responded to my answers.” (Participant 8, female, age 67).*

#### Alexa device design

Participants identified that aspects of the Alexa device’s design made it convenient to access the program.

*“Anyway, it can stay where it is, you know what I mean? I don’t have to transport it around the house and the volume is important that you can adjust the volume. It is kind of like a more-or-less permanent fixture in the house. So that was good- it was inobtrusive and small enough but usable enough if you know what I mean.” (Participant 3, female, age 67).*

*“The positive is it’s there, you can use it as many times as you want, it’s handy, it’s close by.” (Participant 1, male, age 70).*

*“Yeah. I went away to Byron Bay and I only took carry on. I squashed it in there, so it was all right.” (Participant 14, male, age 74).*

#### Ease of use and enjoyability

Participants also found Alexa and Buddy Link enjoyable and simple to use:

*“It’s easy to use. There is a lot of exercises. You can skip what you don’t want. You do what you want and what you need. Those are all pluses.” (Participant 1, male, age 70).*

*“I enjoyed using it. I found Alexa (and the Buddy Link software program) very easy to use.” (Participant 7, female, age 67).*

#### Motivation to participate in the exercise program

The conversation-based functionality of the Alexa device provided motivation to participate in the exercise program.

*“(An exercise program is difficult) to do… because sometimes you get lazy and you don’t really want to do things. But if you’re getting directions and instructions (from the Alexa) along the way, you’re doing it without an effort because it’s gentle.” (Participant 9, female, age 67).*

*“No. I think the thought behind it is good and I can only speak personally for myself that it made me more interested and motivated to do (the exercise program). Because if you’re by yourself all the time and you're not mixing with other people, or in a group situation, and I used to go to group exercises, to keep yourself motivated to do exercises several times a day when you get to the age range that I'm in” (Participant 5, female, age 79).*

*“I did the exercise because she (Alexa) always asks me (do you) want to do it again? So, I did it again, but all the other times when I said I’ll do it again it’s because I was not happy with myself and I wanted to improve it. It’s a really thought through program” (Participant 3, female, age 67).*

#### Social engagement with Alexa

Some participants reported a level of social “human” engagement with the Alexa that supported conversation-based interactions.

*“I would use that device. …I really like this because she’s always asking.., 'Are you ready?' So, you always have to give a response. You can’t just say oh well, I’d like to quickly go and get the bin out or what. You just can’t.” (Participant 3, female, age 67).*

*“(I enjoyed) that someone was talking to me when you live on your own.” (Participant 8, female, age 67).*

Using the Alexa provided a feasible means of engaging with an asynchronous telehealth platform for participants with visual issues.

*“Yes, that’s convenient. Especially now that I’ve had the cataract operated on, it’s quite hard for me to read small print so I’ve got to find glasses which are never far away.” (Participant 15, female, age 74).*

#### Screen and voice compatible reminders

The reminder functionality of the Alexa device delivered using screen and conversation-based interactions provided motivation to participate in the exercise program.

*“The advantage of having it there, knowing that it was going to remind me to do my exercises was a good thing, because I do have a physio app on my phone, but I have to actually go onto it to use it, where this actually would be reminding me. I feel very guilty if I didn’t do it.” (Participant 9, female, age 65).*

*“I thought it was a great way to be reminded and would definitely influence me to exercise more. So I thought it was a very good thing from that point of view.” (Participant 7, female, age 67).*

*“Yeah, I found that it actually made you commit to doing it. Because it would give you reminders, you kind of felt obligated to complete it, you wouldn't just be going …Oh, I'm too busy,…or I can't be bothered….So I found that was really good. It sort of made you commit to it.” (Participant 2, female, age 66).*

### Theme 2: barriers to interacting with the Alexa to participate in the exercise intervention

#### Technical issues

Some participants reported technical issues, such as poor internet connectivity and accuracy of voice recognition, as barriers to participating in an exercise program delivered by Alexa.

*“The voice interaction is very problematic. It does not understand a clearly enunciated yes or no, and I think there’s a reason for that. But you’ll see lots and lots of examples where it just refuses to understand a simple yes or no, or any other command, and given that it’s voice-driven that’s a critical failure.” (Participant 6, male, age 77).*

*“No, the main negative was it may be my (Internet WiFI) connectivity here, I notice on Saturdays it always goes bad for the last three or four weeks, when people have been escaping from Melbourne and bringing their devices down, swamping the bandwidth. Last Saturday it took one session when I waited for it, a good three quarters of an hour. So the “exercise program” doesn't work when your connectivity is not good.” (Participant 11, male, age 71).*

#### Preferences for other existing telehealth modes of exercise delivery

Some participants expressed a preference for their exercise program to be delivered and monitored by other existing telehealth models (such as tablets and smartphones).

*“Yeah. I think I might’ve mentioned that such a program could probably be implemented just as easily and alternatively as a web-based solution, which would just avoid all the annoyance of the voice-based program.” (Participant 6, male, age 77).*

*“I've got my old phone, my present phone, an old tablet, on a really old tablet as well, and plus my laptop. I think it's a much better idea to put it onto the web, honestly.” (Participant 11, male, age 71).*

#### Lack of real-time objective feedback in an asynchronous digital health approach

Participants reported the lack of instant feedback and reassurance regarding correct exercise technique that was not provided by the asynchronous Alexa program.

*“You don’t get feedback, so for somebody who’s not maybe that good at doing their exercises, I don’t know how you would be able to but it would be great if they would get a bit of a feedback on it, whether there’s a physical way you could get somebody to do the exercise on a certain spot and put the camera on a certain spot and then it would be able to measure – or what, I don’t know. Maybe a future version.” (Participant 9, female, age 65).*

*“Yeah, even if it was saying ‘Too fast. Too fast,’ because that’s what I tend to do, everything too fast. Or you know, it might say ‘Just one more,’ you know, that sort of thing. Yeah, I think that sort of feedback would make it even more engaging for people” (Participant 9, female, age 65).*

*“I used to do some of these exercises with the exercise physiologist, I know how you’re meant to do them. But for some people, they probably need a little bit more guidance on some of the exercises. Like I know you do say ‘hold your bum in’ or whatever, but some of them it’s quite important that they do it correctly. So it’s tricky because the first time they do it they need the full instruction and then after that you’re just say reminding them what the exercise is, so.” (Participant 9, female, age 65).*

#### Inadequate attention to patient goals in an asynchronous digital health approach

Participants reported a desire for exercise programs to include information on the purpose of exercise, and to explore and address patient goals, that was not met by the Alexa program:

*“No, it’s always good to have a real human being to deal with. But as a substitute, I think it’s quite good. The other thing was I did get bored. You brought in some more exercises to give some variety and I suggested explaining why we were doing these. I’m guessing that the balance is good for someone my age because falls can be a problem as you get overzealous, sort of thing. But the explanation of the value of what we were doing would be good, the physical value.” (Participant 15, female, age 74).*

*“It’s like when I went to the gym, and I have an instructor there, and he said, “Why do you want to be here? What exercises you want to do? Do you want to lose weight? Or you want to just have more agility? Or you want to have fun or whatever it is.” So it can be – then he went and selected five different programs and showed me how to do it. And then every second day I go myself and I do them myself. Some for the leg and the calf, others for the ankles, others for whatever.” (Participant 1, male, age 70).*

#### Privacy concerns

Participants often chose to turn off the Alexa device between exercise sessions due to privacy concerns.

*“Well I had the camera turned off, so I’m not sure – and because I live alone, there’s not too many conversations going on around a machine, but if you had a private conversation you probably wouldn’t have it in front of Alexa. But I don’t know. If you haven’t turned her on I don’t know if there’s any recording happening anyway. I don’t know. I don’t know of that privacy.” (Participant 12, female age 68).*

*“I have turned off the camera except talking to you now. Yes, I suppose with the camera on I would have issues with that, in general, not from Deakin University, but just from Amazon up there in the Cloud, and who else is on it and so on.” (Participant 15, female, age 74).*

*“It did concern me, because you don’t want to think that you’re being listened to all the time and watched all the time, however it was alright once I found where to turn off the camera.” (Participant 7, female, age 67).*

*“Well, I’m a little bit—I mean in terms of the exercise and stuff not really, but if I had to—like, if it was a different kind of program where it’s involving a lot of private things like medical stuff or opinions about things or whatever I might, yeah, think twice about that because the stuff that it’s Alexa or a Google Home thing where they do—you don’t know where your information is and all that sort of thing. Yeah, I mean I’m a bit, not suspicious, but I’m wary of the devices just generally, but in terms of just data for the exercise it’s not an issue for me. It’s only if it’s asking personal information.” (Participant 13, female, age 66).*

*“Well, I wouldn’t leave it on in between sessions and I wouldn't leave the video on at all.” “It's sort of really amazing when you just have a passing comment to someone during the day, your phone picks it up and then there's an ad about it two hours later.” (Participant 11, male, age 71).*

## Discussion

This nested qualitative analysis identified several enablers to a home-based exercise program delivered and monitored remotely by an Alexa supporting conversation-based interactions in older adults living alone. These findings support the notion that a home-based exercise programs supported by VIPAs are feasible in community-dwelling older adults living alone [10]. Participants reported themes relating to Alexa’s role in supporting ease and enjoyability, social engagement and motivation to participate in the exercise program. Nonetheless, participants reported some barriers associated with the Alexa technology, including technical errors, lack of real-time feedback, privacy concerns, and preferences for other existing digital health modes of exercise delivery.

Our qualitative analyses revealed enablers related to user-friendly voice interaction, reminders, and motivation to participate in an exercise program delivered by Alexa. The enablers that we identified were similar with the only other qualitative study exploring the barriers and enablers to participating in an 4-week non-tailored, home-based exercise program delivered and monitored by a VIPA (speaker only) in 22 healthy participants aged 20–65 years [14]. Participants in this standalone intevention reported the user-friendliness of VIPAs while participating in an upper-limb exercise program as an enabler. There is evidence to support the use of blended care, i.e. a multidisciplinary model of care to increase physical activity behaviour among community-dwelling older adults. A recent systematic review of 13,829 older adults over 65 years found interventions employing a combination of cognitive and exercise strategies were more effective than exercise alone to increase physical activity behaviour [15]. A recent systematic review of 56 studies exploring barriers that prevent ubiquitous use of telehealth reported that 17% of older adults identified hand–eye coordination, visual acuity, and mental acuity as barriers to traditional digital health delivery of part of general care [16]. Conversation-based interactions may therefore be helpful in overcoming some of the challenges of traditional digital health modalities, especially when used during exercise sessions requiring use of the hands (e.g., during upper-limb exercises). Future comparative qualitative studies are required to identify whether determinants of adherence to exercise in older adults participating in a remotely-supervised asynchronous program delivered via Alexa, differ to those for exercise interventions delivered using other digital health tools (e.g., tablets and/or smartphones). Furthermore, comparative qualitative studies are required to identify whether determinants of adherence to exercise in older adults participating in a remotely-supervised prescribed by a single exercise physiologist delivered via Alexa, differ to those for exercise interventions delivered using blended care (e.g., exercise physiologist and psychologist).

The themes of companionship that we identified are supported by previous work examining factors of conversation-based engagement with an Alexa device [8, 9]. Interacting with a ‘new friend’ in the home environment and having someone to talk to in natural language may help alleviate loneliness and improve companionship and mental health. A qualitative interpretative study examining human comparison interaction theories and para-social relationship theory with 12 VIPA users found that factors influencing acceptance and usage of VIPAs included functional and hedonic benefits, such as perceived enjoyment [17]. Furthermore, social characteristics of the VIPA, such as mimicking human-like voice attributes, may elicit a sense of social presence and assist in developing a trustworthy relationship with the user [17]. Therefore, older adults living alone who interact with VIPAs using natural conversation-based interactions may develop a deeper para social engagement.

Some participants in our study reported the lack of real-time feedback in an asynchronous telehealth approach as an extrinsic barrier. There is evidence to support our finding that some older adults prefer to exercise with synchronous digital health support. For instance, a qualitative study nested within a 12-week home-based, randomised controlled trial in which pre-disabled older adults were prescribed a supervised, home-based exercise via video conferencing (mean ± SD age 77 ± 6.9 years) or phone (mean ± SD age 80.1 ± 5.9 years) found the regular help and real-time follow-up using synchronous videoconferencing from kinesiologists as an enabler to help facilitate adherence [18]. Using the Alexa device videoconferencing feature to enable instant feedback and reassurance (e.g., regarding correct exercise technique), once a fortnight for example, may alleviate some of these concerns in future exercise trials delivered by VIPAs. However, telephone calls or videoconferencing rely on synchronous contact with a health professional, which is both time and labour intensive (and therefore costly). There is the potential to develop algorithms based on participant voice responses (e.g., regarding correct exercise technique) that can automate delivery of feedback to participants by VIPAs.

Some participants in our study reported a preference for using other existing digital health tools for exercise program delivery. This may be due to a lower familiarity with using VIPAs compared with traditional digital health devices such as tablets and smartphones. A recent systematic review of 56 studies identifying barriers to ubiquitous telehealth use identified technical literacy (17%) and lack of desire (13%) as the most common barriers to telehealth delivery of care in older adults [16]. Establishing user preference for VIPAs compared with other digital tools, such as smartphone/tablet and web programs, for monitoring and delivering home-based exercise programs should also be considered in future trials.

Poor internet connectivity were identified as extrinsic barriers associated with using VIPAs in some participants in our study. Poor internet connections sometimes precluded the participant from participating in their prescribed exercise program at their preferred time of the day. Adequate internet bandwidth is a critical factor to effectively deliver internet-based digital health approaches [19]. A qualitative study involving older adults who received total knee arthroplasty allocated to either hospital- or home-based programs with videoconferencing follow-up found technical difficulties such as voice/image synchronisation related to inadequate bandwidth as the most common area of dissatisfaction [20]. Nevertheless, this barrier seemingly did not impact our participants’ above average usability (75/100) or mean adherence (115%) to the exercise intervention at 12-weeks [10] as participants often re-engaged with the program at a later time if technical/connectivity issues were experienced.

Participants in our study often chose to turn off the device when not being used for the exercise program due to privacy concerns. Recent findings suggest almost 41% of VIPA users are concerned about privacy, and passive voice-activation background listening has been identified as the main barrier for VIPA users [21]. However, this barrier did not deter our participants interacting with the Alexa to adhere to their prescribed exercise program [10]. Participants in our study reported similar themes to those in a qualitative study of nine VIPA users exploring factors influencing consumer trust with VIPAs. Participants in this study reported themes related to the unidimensional model of trust with the willingness of a VIPA user to be vulnerable to third party data monitoring as long as the VIPA can fulfill an entrusted and important task [7]. Participants in our study appeared willing to engage with Alexa irrespective of the ability of a third party (i.e., Amazon) to monitor or control personal data based on the expectation that the VIPA device would perform an action important to the user (i.e., deliver the exercise program). In addition, our participants reported their VIPA interactions outside the exercise program were primarily adopted for daily routine functions, such as asking Alexa for weather forecasts, and not for tasks where privacy and security may be of greater concern, such as for making online purchases. The built-in camera shutter feature on the Alexa was also appreciated by participants as a means to assure their privacy.

### Limitations

The data were collected and analysed by researchers who were involved in the trial, which may have affected intellectual bias. Furthermore, trustworthiness may not have been achieved as the transcript and codes were not checked and confirmed by the participants in this study. Another limitation may be selection bias because participants who agreed to participate in the trial may have had some positive feelings or previous familiarity engaging with a VIPA.

## Conclusion

This qualitative study nested within a 12-week, prospective single-arm pilot study identified several enablers to a personalised, home-based exercise program, delivered and monitored by an Alexa supporting conversation-based interactions. However, based on this study it is recommended that future exercise intervention studies using Alexa should focus on reducing voice recognition errors, providing real-time feedback (e.g., with regards to exercise technique), and comparing patient experiences of exercise sessions delivered via Alexa versus alternate digital health tools (such as tablets and smartphones).

## Supplementary Information

**Additional file 1.**

**Additional file 2.**

## Data Availability

All data generated during this study are included in this published article and its supplementary 2 Transcription file.
