# Supplementary material for: Barriers and enablers for older adults participating in a home-based pragmatic exercise program delivered and monitored by Amazon Alexa: a qualitative study
Source: BMC Geriatr. 2022 Mar 25;22:248. doi: 10.1186/s12877-022-02963-2 (PMC8953055; doi:10.1186/s12877-022-02963-2)
Supplement: Supplementary file 1 — Additional file 1. [file 12877_2022_2963_MOESM1_ESM.docx]

**Supplementary 1**

Examples of qualitative questions:

- How would you describe your experience during this project?
- What have been the positives of using the Alexa to adhere to the exercise program?
- What have been the negatives of using the Alexa to adhere to the exercise program?
- How easy or difficult was it for you to participate in your prescribed program regularly using the Alexa?
- What prevented you from participating in your prescribed program regularly using the Alexa?
- What enabled you to participate in your prescribed program regularly using the Alexa?
- Are you likely to continue using the Alexa device to adhere to exercise program? If not, why not?
- Do you have any concerns regarding data privacy using the Alexa device to adhere to the exercise program?
