# Supplementary material for: Barriers and enablers for older adults participating in a home-based pragmatic exercise program delivered and monitored by Amazon Alexa: a qualitative study
Source: BMC Geriatr. 2022 Mar 25;22:248. doi: 10.1186/s12877-022-02963-2 (PMC8953055; doi:10.1186/s12877-022-02963-2)
Supplement: Supplementary file 2 — Additional file 2. [file 12877_2022_2963_MOESM2_ESM.docx]

**postS001**

**Q:** We are with participant 001. It’s the 21^st^ of December at 11.00. The first question is, how would you describe your experience during this project?

**A:** Oh, it wasn’t as simple to participate, put it that way. So I didn’t do as much as I wanted for a few different reasons. One of them is once COVID restrictions eased, I started going to the gym myself using different [things] using – especially I enjoyed the hydro. And I was doing hydro and spa and all other things. So I didn’t really need the system and the exercise anymore.

**Q:** So back to the Alexa device, what do you believe are the positives of using the Alexa device?

**A:** The positive is it’s there, you can use it as many times as you want, it’s handy, it’s close by. That’s other plus. It’s easy to use. There is a lot of exercises. You can skip what you don’t want. You do what you want and what you need. Those are all pluses.

**Q:** Great. Did you find it easy that you could activate the device through voice and not through touch? Was that a good thing?

**A:** Yeah, it’s not complicated at all. But put it that way, it doesn’t have too many functions anyhow. I mean, it’s really simple, very easy. I wish [had] more functions like connection to another screen. I don’t know. And we’re going to try to see if it’s – I’m not sure. And [Jack] couldn’t answer me regarding can we use it like Google for Chromecast. Because I need one right now and I might use this one if it works. But I’m not sure if it works. Like the other one, the Google I remember there is – once it’s connected to the internet, to the website or internet, it’s just a matter of asking Google to connect it. The position, if it’s a bedroom or whatever, [Mina] bedroom or family room, which TV and whatever. I don’t know if it’s Alexa can do this sort of thing.

**Q:** Yeah. I’m not sure either. But as far as the Alexa, did you use the Alexa for reminders or checking the weather? Or did you use it for any other function?

**A:** The weather is always written there, but yeah, a few things, yes. But I’m not sure is it – once it’s connected to the internet, supposed to function the same as Google, isn’t it?

**Q:** It is, yeah.

**A:** I mean, it’s what you do really. It’s a means of communication with the internet. But does it have the same program, that’s another thing. The same language. I don’t know.

**Q:** Yeah, I’m not sure. Did you have any -

**A:** Yeah, you have properly to ask it. To ask Alexa if they have the same language or which language to use to do certain things.

**Q:** Yeah, exactly right. Were there any other positives of using the Alexa device to deliver the exercise program?

**A:** I don’t think – I don’t know really. I mean, it’s really handy, really – the size is really reasonable. Small. It’s not heavy. You can move it. I’m not sure if I can use it without the power. I don’t think so. Is it?

**Q:** No, that’s correct.

**A:** If I disconnect to the power, that’s it. I lose everything.

**Q:** That’s correct. Yep.

**A:** Now, has it got a camera? Can I use it for Skyping?

**Q:** You can. Exactly. No, you can.

**A:** I can?

**Q:** Yep.

**A:** I can. Or Zoom or all these programs. But I have to download the program, is that correct?

**Q:** That’s correct. Yep.

**A:** And is it easy to download? I don’t know how to download anything.

**Q:** Yeah. Oh look, I -

**A:** It’s just -

**Q:** I can help you run through that after the interview.

**A:** Okay then. That would be good. That’s very important, actually. Because you have to [stand there -] and I think would be good idea if you can send email to all – that’s – they would appreciate you, appreciate the system, appreciate the research program altogether if you can send us email with how – what you know about the function of the system itself.

**Q:** Absolutely.

**A:** Everybody would appreciate that.

**Q:** Absolutely. Sorry.

**A:** And – sorry, okay.

**Q:** Sorry, excuse me.

**A:** So if you – sorry, can I talk?

**Q:** Yep. No, that is good.

**A:** It doesn’t have enough – and I know why. I mean, you haven’t planned it for this but you don’t have enough exercise to choose from. Or maybe you have enough, but I don’t[ inaudible]. I heard from [Paul], from [Jack] that you have more exercises that you didn’t include it.

**Q:** Yep. So you would have liked to actually been able to choose your own exercises. Is that correct?

**A:** Sorry, you’ve been cutting off and I couldn’t hear you.

**Q:** So you would have liked to choose your own exercise. Is that correct?

**A:** Yes, yes. With your help. Like you could ask me – ask the client like what do you like, why you want the exercises. It’s like when I went recently to the gym, and I have an instructor there, and he said, “Why do you want to be here? What exercises you want to do? Do you want to lose weight? Or you want to just have more agility? Or you want to have fun or whatever it is.” So it can be – then he went and selected five different programs and showed me how to do it. And then every second day I do myself and I do them myself. Some for the leg and the calf, others for the ankles, others for whatever.

**Q:** So what about the actual exercise snacking program? So the exercise intervention was delivered in shout bouts frequently through the day. Did you find that good?

**A:** Sorry, the line or – the website or the internet, I’m not sure, is very slow.

**Q:** That’s all right.

**A:** But your wording has been cutting off and delaying. There is a delay.

**Q:** Okay. Did you like -

**A:** So it’s not synchronised.

**Q:** Did you like the -

**A:** [inaudible]

**Q:** Would you be willing to purchase the Alexa device at your own cost?

**A:** I didn’t hear you but did you mean purchasing? I didn’t know what you say.

**Q:** Yeah, that’s cool.

**A:** I didn’t hear it at all, but I assume willing to purchase. No.

**Q:** Do you have any issues – sorry.

**A:** Because I am already in a gym. I’m already paying $17 a week, and that’s discounted instead of $22 or $24, I’m paying $17 because I’m a pensioner. And that’s where if you’re maybe doing discounted fee for concession and pensioner, I think maybe would get a portion of client. And if you have more – if the system got more use, like Chromecast, I think people would buy it. So it will be multiple systems.

**Q:** Okay. Do you have any issues -

**A:** I think I would buy it for different – for another reason. For connecting to my TV and having the Netflix and whatever.

**Q:** Do you have any issues with data privacy?

**A:** Sorry, I can’t hear you at all. I lost the – I cannot say what you are saying.

**Q:** That’s all right.

**A:** I cannot hear what you are saying.

**Q:** Do you have any problems with data privacy?

**A:** I don’t know. I don’t know if having a camera there and when the system is not used, we hear - we hear in computer and laptop and whatever there are hackers or there are people or somewhere they can spy on you. And that’s worrying people that maybe on the camera – and do you know, there are some gadgets now that you put something on the camera so nobody – when it’s switched off, nobody can see you. Even if you have – if the system is created with a flip – something you put in and you – actually, this is something I haven’t seen anywhere with camera now, with devices with camera, if you have something to slide.

**Q:** Well, the Alexa has those.

**A:** Do you understand what I’m saying?

**Q:** Yeah, well -

**A:** A slide to cover the camera. I haven’t seen it yet. And maybe you can be the first one to do it.

**Q:** The actual Alexa device has the slide.

**A:** Something that you can slide in this way or a switch to go -

**Q:** No, that’s good.

**A:** - and you can cover the camera. I think you will be the first one to do something like this. And of course [there’s even inaudible]. But if you can get that advertising promotion or advertising that – or marketing that you’re the first one to do it. Oh, I think you’d do a lot of business, because you are invention [genuituity] and innovation. So the company can see that you are – you can be ahead of others.

**Q:** Yep. Do you have any other comments regarding the project?

**A:** Not really. I haven’t lost anything. You are not really – you didn’t really have – I didn’t sign any contract to if I don’t do it I’ll be punished or whatever. I would have loved to see more rewards to be honest. If I have the reward that if I don’t do it I don’t get that reward, I would have done it more. So you know, we are human beings, and that’s some – I know you didn’t have enough money to spread around, but I was with Deakin or Monash or – I don’t remember which university. They were giving $200 supermarket vouchers for participating in diabetes research. Of course you are controlled by the grants you’re getting, but this is always something you can let the people sponsoring you know about it. To be more spread around, you have to put more money in.

**Q:** Yep, that’s correct. Yep. Any other comments before I stop the interview?

**A:** No.

**Q:** Okay, I’ll stop that now.

**postS002**

**Q:** So, it's the 11^th^ of December at 3:13. We're with participant … and the first question is how would you describe your experience during this project?

**A:** Yeah, I've experienced, well, overall quite positive. It was just a shame that I wasn't able to complete the entire study.

**Q:** What would you believe would be the positives of using the Alexa device to deliver the exercise snacking program?

**A:** Yeah, I found that it actually made you commit to doing it. Because it would give you reminders, you kind of felt obligated to complete it, you wouldn't just be going "Oh, I'm too busy," or "I can't be bothered." So I found that was really good. It sort of made you commit to it.

**Q:** Any other positives?

**A:** Yeah, well I always liked the fact that it was flexible enough that if you weren't able to deliver the exact time of the reminder, you could always just pick up and do it a little bit later or a tiny bit earlier to fit in with your schedule.

**Q:** Did you find any negatives of using the Alexa device to deliver the exercise program?

**A:** Yeah, I did find a few in that I found it was a little bit inflexible with regards to if you were interrupted by a phone call or someone knocking on the door during the actual session, that you couldn't actually pause it. You'd have to get right out of it and then go back into it. There were also a few access problems where it would freeze or drop out and the other thing was sometimes you would give the incorrect. I'd sort of find I'd go off with the pixies a little bit sometimes because it'd take a while to get into it and then I might answer yes instead of no or no instead of yes and you couldn't correct it. But then yeah, that was I found a problem. I thought maybe there could've been some extra prompts to make it make a little bit more flexible.

Yeah, and the other thing I found, I tended to get a little bit bored. I found it took a while to get into it, so Alexa would sort of say, you know, like give you the introduction and then the exercises would be shown to you. And once you'd done them a few times, I think you could actually - could've perhaps condensed it a little bit so that it was a little bit faster to get into each exercise. Maybe skip instruction or something if you knew what you were going to do.

**Q:** Yep. No, that's really good feedback. Any other negatives?

**A:** No, I don't think so. I think that was all. That was enough, wasn't it?

**Q:** With those negatives, what did you do to overcome those? Did you just wait for the freezing to subside or did you -

**A:** Yeah, generally if it froze I would sort of get back out and go back in. But I found sometimes that it would miss - it would skip the actual exercise that it had frozen on and move on to the next one.

**Q:** Yep. How easy or difficult was it to set up the Alexa device?

**A:** It was very easy. Yeah, no problems at all with the actual set up.

**Q:** Would you be willing to purchase the Alexa device at your own cost if you were not given one?

**A:** Yeah, I think so. I think I would probably use it for a lot more. Because I haven't been well, I haven't really gotten into all the different functions that it would provide. I haven't really spent time researching what else it will do.

**Q:** Yep. So you haven't used it for reminders or the weather or anything like that?

**A:** No, I haven't. No, I was sort of basically only using it for the exercise program.

**Q:** Yep. Do you have any concerns regarding data privacy using the Alexa device?

**A:** Yeah, probably a little bit. You sort of feel as though it's always listening in but I think when you look at it, we've all got smart TVs and smartphones and I think they do a bit of that as well, so it's a bit of a moot point, isn't it?

**Q:** Did you modify your behaviour at all? Did you, for example, turn off the camera or the voice or actually turn it off completely?

**A:** I did turn off the camera and while I was mostly - or when I was at my worst, my sons were working from home [and] they actually turned it off completely because they were doing a lot of confidential stuff workwise.

**Q:** Yeah.

**A:** Plus it was yelling reminders at them all the time as well.

**Q:** Do you have any other feedback? Any other comments you'd like to make?

**A:** No, I don't think so. I think overall it's a very good program but I do think, yeah, perhaps it could be streamlined a little bit. I kind of felt the exercises that were on there were probably for people perhaps older and less physically active than I am. So if I - yeah, perhaps the exercises, you know, if I was to go on with it, I would want to do perhaps different or a little bit more condensed rather than spread out with the slow instruction side of it.

**Q:** Yeah, that makes sense.

**A:** Because I get bored and started checking emails and stuff like that and would almost then miss the start of the exercise, have to leap out of the chair.

**Q:** Yeah. Perfect. Anything else?

**A:** No, that's all I can think of.

**Q:** Great. I'll stop the recording.

**postS003**

**Q:** – of December at nine o’clock. We’re with participant 003. The first question is how would you describe your experience during this project?

**A:** Well, it was a good experience. I’m glad I took part.

**Q:** Perfect. What’s would have been the positives of using the Alexa device to deliver the exercise snacking program?

**A:** Positive about having the device? Well, look I did - when we couldn’t leave our house, well very - I mean with all the restrictions during Covid-19, it was convenient to have a small, inobtrusive device and not - I mean when I did Zoom classes I always had to transport my desktop to an area where I can do the exercises. So that was - and then after a while, I mean initially I had to look exactly how you were doing the exercise, so I had to go a bit closer but once or twice, I mean I had to look at it again because I had forgotten. Anyway, it can stay where it is, you know what I mean? I don’t have to transport it around the house and the volume is important that you can adjust the volume but you can do that on a desktop as well. It is kind of like a more or less permanent fixture in the house. So that was - I thought that was good, it was inobtrusive and small enough but usable enough if you know what I mean?

**Q:** That’s really good feedback. Any other positives that you can think of?

**A:** Well, it’s the main things. I can’t think of anything else really. I mean to be honest I’m not - I didn’t use it for anything else apart from initially I was curious and I thought let’s see what else I can do. I think it looked like a device that’s what all of my friends, especially the ones who grew up in Australia, what they’re into, they are listening a lot to the radio and they have the radio on as the background noise. Well, I don’t. I didn’t work with radio you see and so I only used it for the exercises. I’m not a radio person. I like the internet but you have to grow up with it I think and I didn’t use it. So, I mean whatever it came up - initially I read it because I thought that could be interesting so I checked in a bit but I only did that for a week or two and that’s it. And then I thought this is, look, I don’t want to cram my day.

My day is so full already and I don’t need more entertainment. Definitely not entertainment, my life is funny enough. And anyway, I didn’t need all this. It was good for what it did and that’s it. So, I could use it again for that kind of purpose but I mean there’s some conditions. Well, how it has to be - because you see in a Zoom class you can just kind of check out if you know what I mean? And nobody would realise but here you couldn’t really, but that’s a different question I suppose. Anyway, I have only used it for the exercises, 95% and that’s it, for the snack exercises thing.

**Q:** Did you find any negatives using the Alexa device?

**A:** Well, we sorted that out because when it came with the reminders. But you see that, well you obviously can change that. And I hate reminders because I just don’t know, look, I’m a very - if I commit to something I usually do it unless something really overrides it and then I can’t help it. But I mean I just know when I have to do stuff.

**Q:** So, no real negatives?

**A:** So, I didn’t, and I thought it was annoying the way they set it. But look, you fixed it so then it was no longer there so that’s fine.

**Q:** Yep. So, the reminders were annoying but there was nothing else that was really a negative?

**A:** Well, I don’t know if it’s a different question but you see I really liked it and I thought about it this morning. I don’t like predictability because it is, well it’s - look, there are places where it’s good to have predictability but that comes with experience and - usually. Because I think you can easily influence it how you put the exercises together. So, when I know after the - what’s that the squat and leg lift, that’s always coming with the what’s it called? The lunging to the side isn’t it? And I mean I thought that boring because I like it - I mean there’s no technical reason for it in my opinion. Well, for example in yoga you can’t do complicated things if you haven’t warmed up your body but because it’s a snack exercise there’s in my opinion no reason why you couldn’t just go crazy. And I mean mix it up every day, every time it comes.

I mean the generator, I mean I know the generator is to do that kind of thing but I don’t know obviously how that works from your side. And I like the unpredictability, you know what I mean? Because it’s only to be on the floor or next to a chair or kitchen thing, bench, in the house so that’s all good. I mean it’s not something I have to do, walk around the block. Well, I would like that because, you know, so it’s - I think in my opinion it would be nice because it’s a surprise and I like things that are completely - yeah, it’s new and you don’t kind of yeah, you can easily do it I think. You can organise it in a way that’s more exciting and more stimulating.

**Q:** Any other negatives at all that you would like to mention?

**A:** No, I kind of basically I can’t - No, I mean I kind of knew I kind of committed to four times and if I didn’t do it, well then there definitely was a reason why I didn’t do it and if you ask me and I can look at my diary what I did. Well, I can maybe I cycled a long distance or I swam or did Pilates and then I was worn out. But otherwise, I did it, I mean there was no other reason. No, I can’t think of the negatives and because it really helped and that’s what it is about. I mean when - you wouldn’t have - I mean I wouldn’t want to commit to four times a day, that would have been oh my god. It’s the amount and the intensity that’s important I think for the whole program.

**Q:** Are you likely to continue completing the program after?

**A:** I would use it, I would use that device. Yes, I thought about that and I would. But you see, I really like this because it doesn’t really let you - and I - it’s really well thought through because she’s always asking or the device always asking, “Are you ready?” So, you always have to give a response. You can’t just say oh well, I’d like to quickly go and get the bin out or what. You just can’t, you’ll not make it. I would use the device, I thought about it but you see I really like this because it doesn’t really let you, it’s really well thought through because she is always asking on the device are you ready? So, you always have to give a response. You can’t just say I need to quickly go and get the bin out. You just can’t. This morning I was trying to do something because I’m baking bread here and I had dough on my fingers. And then what the heck do you do? You can’t really do exercise with dough on your fingers.

And well so, I think I started then again, I don’t know, I did the exercise because she always asks me want to do it again? So, I did it again, but all the other times when I said I’ll do it again it’s because I was not happy with myself and I wanted to improve it. It’s a really thought through program and it’s not - because it’s not something you can just listen to it and kind of do your own thing and be on the phone or cook something. You can’t do this because it always engages you. It’s tricky but it’s good, I mean why would you do it otherwise? It’s not a waste of time. And especially if I pay for it, you know, now it was a freebie but if I paid for it, where sometimes I think oh my god, why do I do this, you can just let it go. Anyway, I thought it was thought through, you know the dynamics of the brains as well.

**Q:** Well, that's my next question. So, would you be willing to pay for the device if you were not given it for free?

**A:** Well, how much would it be? You see if it’s -

**Q:** $100, $150 I think.

**A:** You don’t know that yet or what?

**Q:** No, it’s $150 the device, $150.

**A:** For the device?

**Q:** Yep.

**A:** So, but does it come with the exercises?

**Q:** Yeah, it would, yep.

**A:** For how long do the exercises go?

**Q:** Indefinite, it’s hypothetical so obviously you’re going to - the device is free but we’re just trying to ask the questions to see if other people would be willing to but yeah, the exercise would be ongoing, it’s just the initial cost of the device which would be $150.

**A:** Well, if that’s ongoing and it is structured, I think the structure - is very important I think. If you just kind of run the thing like a disc or like a cassette in the older days you wouldn’t do it. It’s only because it’s interactive and it’s really interactive. You basically - if you don’t respond when she says, you know, that device does something. It actually turns off if you don’t respond at all. I mean it goes back to the time, it reissues the time because I had somebody who sometimes called me but I mean that didn’t happen very often. I usually - it just didn’t happen. I was lucky because I said look, I’ll return the call. Because I just didn’t want that because I had already done half of that thing. And I thought no, I want to continue because otherwise I don’t get this checkout card here, you know?

No, I think it’s - if it’s $150 for whatever, I think that is a good price, you know, what I mean is you get all of the exercises. And you can have influence on how the exercises are structured, that you can say, “Look, I want something - put something - and there is kind of like a feedback loop that you, special conditions that happened in your body, I mean it’s not a real consultation but I mean to kind of self-advise what exercises to take out and maybe not use. And well, if it’s an exercise-focused consultation, I think that’s a good thing. And the price is not too bad either, you see, if you look at how much you pay. I mean the physios, and – to be honest, I’ve had from, let’s say 15 physio I’ve had, I think two were good.

So, I mean, they kind of helped me to improve, where the others, they just give you something like take a tin out of the cupboard and - but there is no - no, I mean not good enough. I mean I had seriously somebody who came home when I got my hip replacement, and she drew little sketches to help me out and she watched every movement. With her I learned stuff. So, she was number one, I mean seriously she was so good and I - that’s actually, I thought see if you can’t move to have somebody in-house, I mean she came I think five times but it felt like ten times at least because she was so good. If I got 20 times to somebody and it doesn’t help, what’s the point? So, I think it’s good but I mean, it has to be supplemented, there has to be something else complemented to make it rounder and more successful, I think.

**Q:** It hast to be monitored?

**A:** Yes, definitely, otherwise you - look I could be sloppy with some of the exercises and well, and it’s not helpful. Maybe it just makes what I have, my condition worse.

**Q:** My last question is do you have any issues with the data privacy, using the device?

**A:** Well, you said - well actually I’ve had things that I’ve got scammed because of my data and somebody - thank god I got help about that. But otherwise - I mean what is recorded? My answers are recorded isn’t it? I mean the yes? Well, well you could definitely find out from my yes that it’s not a native yes. I mean, it’s an ethnically enhanced yes. I don’t know, I have no major issues. I mean well people, if you played that thing in my neighbourhood people would know it’s me because there’s not many Germans in this area.

And some people may know me, especially in the physical area because what I’ve done, I’ve had lots of friends who are quiet, they are still active, you know? I mean cycling, definitely cycling because that seems to be the thing that you can do until you are 80. See what we can do. But I mean walkers, well that’s - all this becomes less because walking, I mean especially long distance, that’s a bit of a challenge, especially if you carry stuff.

**Q:** Do you have any other comments? Any other final comments?

**A:** No, I think I’ve said. Because we had this connection in between which was good so I mean I wouldn’t have liked it at the beginning and end so I’m glad there was something in between as well because then I could, it felt like a debrief. It was good. I’m glad I was in it and I could affirm, not confirm - when I could fear that what I believed in, that’s true, because if you have to - if you want to be fit you just have to keep it up. And sometimes the amount is not necessary, it’s more the regular thing and it’s not that you suddenly go 50 km, walk 50 km, that’s not required.

**Q:** Perfect, yeah.

**A:** No, I think it’s - it’s a good thing, yep.

**Q:** Good, all right. Well, I’ll stop the recording now. Whoops. Turn off.

**posts004**

**Q:** So it’s the 23^rd^ of December at five o’clock. We are with participant 004. The first question is: what do you believe were the positives of using the Alexa device to deliver the [exercise tracking] 00:00:13 program?

**A:** I think positive.

**Q:** Okay.

**A:** I just [unclear]–

**Q:** Okay. So we were –

**A:** – [that you’ve just experienced].

**Q:** Yeah. So with the device, did you use it for any other means apart from the exercise? So, setting reminders, checking the weather, anything like that?

**A:** Well, I started to, but every time I ask for the weather, it’s 12 hours old; news, 24 hours old. And the Irish music that I like, it only plays about 50 songs on rotation.

**Q:** So they were positives. What do you feel were the negatives of the actual exercise program?

**A:** Not hard enough.

**Q:** Not hard enough.

**A:** [Unclear].

**Q:** Yeah. What about the short bout of frequent exercise? How do you find that?

**A:** [What]?

**Q:** The short bout of frequent – how the exercise sessions were dispersed throughout the day.

**A:** I didn’t – [my stuff]– what I did, I spread it when I could do it, not a regimented –

**Q:** So what about the actual device itself? What were the negatives of that? So you experienced a few dropouts. Was there anything else?

**A:** [Not a few].

**Q:** Quite a few?

**A:** Quite a few.

**Q:** Yeah. Was there any consistency between those dropouts?

**A:** [Unclear] it would not recognise you 50% of the times.

**Q:** There’s no ways or means to work around that?

**A:** Oh yes. [The ways of doing] was to go back home and start the whole thing all over again. And then what you [unclear] experiences – you’d get to the fourth exercise and again, she’d probably not recognise yes and then she’ll drop out.

**Q:** Did you like the [act] that the device was voice activated, not touch screen? Did you find that helpful at all or not so much?

**A:** [Unclear]. The touch screen might have been less ambiguous about my yes answers.

**Q:** I guess the reason why that – the questioning is that some people liked the act of it being handsfree. You’re not reliant on going to say smartphone or touching or swiping to activate it. But that wasn’t really the case for you?

**A:** No.

**Q:** No. Did you find that the frequent exercises were getting in the way of your day-to-day activities or not so much?

**A:** Yes, they were.

**Q:** They were.

**Q:** No. Okay. Would you be willing to purchase the device at your own cost?

**A:** No, [unclear].

**Q:** Are you likely to –

**A:** I would ask you where can I send it back?

**Q:** We don’t have capacity to do that, unfortunately. The next question is are you likely to continue with the exercise program after this project is finished?

**A:** No, [unclear] [own program].

**Q:** Yeah. Okay.

**A:** [Unclear] calories in a session three times a week.

**Q:** Do you have any issues regarding data privacy?

**A:** [Unclear].

**Q:** Do you have any other comments you’d like to make regarding the positives or negatives of the project?

**A:** No, I think I’ve covered that.

**Q:** Okay. All right. Well, very happy to leave it there. I’ll stop the recording.

**postS005**

**Q:** It’s 10.00 o'clock. We’re with participant 005. It’s 15 December 2020. The first question is: how did you find your experience in the project?

**A:** I found it interesting and motivating, though the last few weeks, Alexa has not been as helpful as she was initially. And I found that some of the exercises that they put together were strange combinations – to do a sit-to-stand with a calf raise, a squat, and then a squat with a leg raise, one after the other in a set of four exercises, I think is not a good enough variation.

**Q:** Okay. That’s really good feedback. So, we’ll just touch on the positives first and then we’ll go back to the negatives. So, what do you believe were the positives of using the Alexa device to deliver the exercise program?

**A:** I found having someone to watch and to call you at certain times to do the exercises more motivating than to have to remember to do them by yourself.

**Q:** Okay, great. Any other positives of the device?

**A:** No. I think that’s a big positive.

**Q:** Absolutely. Did you use any of the other functions apart from the exercise? So, reminders or anything?

**A:** I didn’t use them perhaps as much as I could have, but I did get used to asking Alexa for the time, the temperature on that day and in future days as reminder to do certain things. So I think there’s a big opportunity. It’s just that someone like me is not familiar with one of these devices to get used to it and to use the full functions of the machine.

**Q:** Yeah, makes sense. And did you find the positive of a pretty small device with a video function. Did you find that a good thing?

**A:** Yes, I haven’t used any other device like this. I suppose a bigger screen would be helpful. But I don’t know that, because this is the only size I’ve used.

**Q:** Absolutely. And do you like that it’s voice activated, that you're not reliant on swiping or touching?

**A:** Sorry, I didn’t catch that, Paul.

**Q:** Did you like that you could activate that it through voice and it’s not traditionally the swiping or touching that smart phones are reliant on?

**A:** Yes.

**Q:** Great. So you touched on the negatives a little bit previously. So, we’ll just go back on that. So, what do you believe were the negatives of the Alexa device delivering the exercise program?

**A:** And that even though I think – I don't know what your thoughts are – but my voice is fairly clear and strong, and it got worse in the last few weeks that Alexa would regularly, in fact frequently, say to me, “I did not get your response.” And I could tell when that was going to happen by where the lighter blue line was on the screen.

**Q:** Yeah, okay. That’s really interesting. Any other negatives?

**A:** No, not that I noticed.

**Q:** Yeah, absolutely. Would you be willing to continue using the Alexa device to deliver the exercise snacking program after essentially today?

**A:** I missed a number of words there Paul. I heard you say would I be willing to continue using the device and my answer to that is yes. But after that I don't know what you said.

**Q:** No, essentially that, so that’s my question.

**A:** Yeah, I think it would be wise. And I did ask Jack I think when I was speaking to him last time, was it likely to continue or what was going to happen next. And he said he hadn’t heard at that time.

**Q:** Yeah. So from my understanding is that you’ll essentially – you’ll obviously get to keep the device and the exercise program will continue. It just may not be as monitored as frequent as we have been.

**A:** Okay. So, the range of exercises would be available on the screen. Would you select your own times or you’d still be prompted at certain times, or -?

**Q:** No, you can select your own times then.

**A:** Okay. I think it’s good for you to have times and do them at that frequency regularly. Not be too random. And all I'm commenting on is for me.

**Q:** Yeah, absolutely. So you like the reminders?

**A:** I do.

**Q:** Good. If you were not given one for free, would you be willing to purchase the Alexa device at your own cost?

**A:** I missed a couple of words there Paul. If I was -

**Q:** If you were not given the Alexa device for free, would you be willing to purchase it at your own cost?

**A:** Depending on how high that cost was. If it was something that I could afford and manage, yes. That would be the only thing that would stop me, if the cost was prohibitive and it was out of my price range.

**Q:** Yeah, that makes sense. Do you have any issues regarding data privacy?

**A:** No, not really. I hadn’t given it a lot of thought, but depending what data you’ve got there and I don’t really know that. But if it’s someone watching me exercise, good luck to them.

**Q:** Do you have any further comments you’d like to make?

**A:** No, I’m delighted, and consider myself very fortunate that I was able to get the device and use it, and get assistance to use it. It really made my day. In the last few months we haven’t had too many wonderful days, but that one was one of mine.

**Q:** Oh, that’s really good to hear. Perfect. Well, if you’ve got no other further comments, I’ll stop the recording.

**A:** Paul, where does the feedback go to? I assume that it goes past you to other people. Is it people who are doing the trials, or people who provided the test? And where does it -

**postS006**

**Q:** Today is 15 December at 12 o’clock. We’re with participant 006. First question is: how did you find the project overall?

**A:** Yep. It was okay. I didn’t have any problems with it. It went on a little too long, perhaps. You know, I always start to get sick of it after a while, but yeah. No real complaints.

**Q:** Great. What do you believe were the positives of using the Alexa device to deliver the exercise snacking program?

**A:** Well, I think I explained that in my email to you. The Alexa device has a lot of problems; seriously, almost every second response it refuses to understand. Having this as a voice-based program I think has got whiskers on it.

**Q:** Okay. We might touch into the negatives, but we’ll start off with the positives, first. Were there any positives?

**A:** Sorry. You’re breaking up a little bit, Paul.

**Q:** Were there any positives of the Alexa device?

**A:** I didn’t find any, no. I find them a bit of an annoyance, quite frankly. It’s not something I would buy. Yeah. I think I alluded that; I think they’re a little bit of a solution looking for a question.

**Q:** Yep. You prefer the traditional touching and swiping Telehealth side of it?

**A:** Sorry, you broke up there, Paul. Say that again?

**Q:** You don’t mind using a laptop, you don’t mind using your phone? You much prefer using those kind of devices than a voice-activated device?

**A:** Absolutely. Yeah. I think I might’ve mentioned that such a program could probably be implemented just as easily and alternatively as a web-based solution, which would just avoid all the annoyance of the voice-based program. It just seemed to me that the program was a little bit odd, having started up the exercises in the morning you’re then asked, “Do you want to start?” Well, of course I want to start, I’ve just started it up. Sorry, getting away from the negatives. Yeah. I think it was useful in that you could show what the exercise was about before you undertook it, that’s fine; so having the visual capability is fine. But other than that, as I said, probably something I wouldn’t use myself if I had the choice. But I persisted throughout, I went through it all.

**Q:** Yep. So you wouldn’t use it for reminders, you wouldn’t use it for checking the weather, that kind of stuff? You’ve got your other devices to do that?

**A:** I have millions of other ways of doing that and, no, I would definitely not rely on it at all, I’m afraid.

**Q:** Yep. That makes sense. Were there any other positives that you found?

**A:** Not really, Paul, I’m afraid to say. It works up to a point. It’s tolerable. It does prove the point you can deliver a program like this via such a device, but that’s about as far as I’m prepared to go really.

**Q:** Yep. What about the negatives? We’ve touched on that a little bit.

**A:** Sorry?

**Q:** The negatives of the device.

**A:** The negatives. Well, basically what I said before. The voice interaction is very problematic. It does not understand a clearly enunciated yes or no, and I think there’s a reason for that. I think there’s something wrong with the timing so that it asks a question and then there’s a second delay before it’s prepared to receive an answer. If you respond before that, even though it’s a clear yes or no, it doesn’t understand it. Worse than that, it gets itself into a loop and doesn’t do the sorts of things that you would expect it to do. For instance, when it gets into a state where it says, “Do you want to wait or continue?” And you say, “Continue,” it freezes. If you say, “Wait,” it just goes into a loop. So the kind of expected responses that you would expect just aren’t there. There’s something sus with the programming. It hasn’t considered at each of the decision points what’s an appropriate response and provided a pathway for each of those appropriate responses. Anyway, I’m sure that’ll improve with time.

**Q:** Yep. No, that is really good feedback. Thank you. Were there any other negatives of the device?

**A:** Not really. I thought some of it was quite superfluous, like, “This is the ABC exercise. The next exercise is ABC. ABC is the next exercise.” That kind of script is a little odd. Don’t know why it was there. It just needs trimming, yeah. It just needs a bit of a trimming and cleaning up.

**Q:** Yep. That makes sense. I’m just having a look through your email now to see if there’s anything else.

**A:** Sorry, say again, Paul?

**Q:** I’m just looking through your email now to see if there’s anything else. Is there anything else you’d like to add regarding that?

**A:** No. I think it’s all explained in there. I’ve given multiple examples and there’s lots of examples in the remainder of the diary which I’ll send you shortly after the program’s finished. But you’ll see lots and lots of examples where it just refuses to understand a simple yes or no, or any other command, and given that it’s voice-driven that’s a critical failure.

**Q:** Absolutely. What about the exercise program itself, the exercise snacking program? Did you find that of benefit?

**A:** I didn’t understand any of that, Paul. Say it again?

**Q:** Good. I think you’ve probably answered this question, but would you be likely to continue with the exercise program after the project’s completed?

**A:** You’re breaking up, Paul. Sorry.

**Q:** Would you be willing to continue with the program after it’s completed?

**A:** What would that involve?

**Q:** Would you continue with it, in an ongoing manner?

**A:** Well, once the official program finishes I think I’d like to keep doing a number of the exercises just by myself for my own personal benefit. Certainly, yes.

**Q:** Excellent.

**A:** They’d probably be, as I said, in the moderate column because the gym that I used to go to hasn’t started up again. It went down the gurgler because of the pandemic, but I’ve found a new one and that will be my main focus for fitness. But I’ll definitely keep the programmed exercises in mind and do them from time to time. Yep.

**Q:** Excellent.

**A:** Is that what you meant?

**Q:** Yeah, absolutely. Yep.

**A:** Yep.

**Q:** Do you have any issues regarding data privacy?

**A:** You’re out of sync again, Paul. Sorry. Your mouth is not saying what I’m hearing.

**Q:** Do you have any problems regarding the data privacy using the device?

**A:** Yeah. As soon as I finish the program, I turn it off. No, I’m really not comfortable having a device like this with an always-open microphone and camera in your home. As far as I’m concerned, that’s not on.

**Q:** Yep. That’s fair enough. Would you be willing to pay for the device out of your own cost?

**A:** No. Wouldn’t even think about it.

**Q:** Yep. No, that’s all right. Do you have any other comments you’d like to make?

**A:** I don’t think so. I think most of them were summarised, Paul, in that email. There’s really not much more that I can say. So unless you have any further questions, no.

**Q:** No. I’m happy to stop the recording.

**postS007**

**Q:** It is the 15^th^ of December and it’s one o’clock. We’re with participant 007. So overall, how was your experience with the project?

**A:** I enjoyed using it. I found Alexa very easy to use and the program relatively easy to use. I was a little bit annoyed with it at times because of the way it was scripted, it felt like it was a bit time wasting when the exercises could have been a bit more joined together to the next one, rather than going through the same sort of set of questions or spiel over and over.

**Q:** No, that’s really good feedback. We’ll just start off with the positives. So what were your overall positives of using the Alexa device to deliver the exercise program?

**A:** I thought it was a great way to be reminded and would definitely influence me to exercise more. So I thought it was a very good thing from that point of view.

**Q:** Good. Did you like the voice activated non-swiping or touching aspect of the Alexa device?

**A:** Yes, I found that extremely useful because at times it might have reminded me at a time where I had my hands in the cake mix or something. So it was extremely beneficial to have that voice activation.

**Q:** Great. Did you use it for reminders or checking the weather or anything else?

**A:** Yes, I did. And I also used it for cooking instructions and I used it for playing music on Amazon music. So yeah, I got good use out of it. It was wonderful.

**Q:** Good. Any negatives of the device?

**A:** Negatives? No, I think any negatives I had were really because I wasn’t slowing down enough. I tended to give instructions like, “Alexa play some music,” all in one breath, whereas it didn’t recognise it unless I went the name – I don’t want to say it again and upset what we’re doing, but the name, break, and then the instruction. Once I learned to do that, I didn’t have a problem.

**Q:** Perfect. Any other negatives?

**A:** Not that I can think of. I’m sorry to do not be able to elaborate, but it seemed fine.

**Q:** Any other comments around the exercise program?

**A:** Well, I think it would have been nice – do you mean the program itself or the scripting of the program? Or the description of the exercises or what?

**Q:** Yeah, no, both. All of that.

**A:** Okay, I think the scripting tended to be very monotonous, with, “The next exercise is – here is the next exercise. [Paul] is going to demonstrate the next exercise. Are you ready to start the next exercise?” And you’re sitting waiting, waiting, waiting, waiting. So I think that really needs looking at. I think it would have been improved if we had an explanation of the correct way to perform the exercises. I know you did a bit of an overview, but more like this exercise is focusing on your arm muscles. Make sure you’re standing at this distance or this height or whatever. And then the next one, this one is focusing on your thigh muscles. Maybe do it this way. Just so that you had a bit more focus on the way the exercise is benefiting.

**Q:** Yep. Anything else?

**A:** Let me see. I felt a little bit held back because I’d been instructed not to do additional exercising, so I really made sure that I didn’t go for those long walks or didn’t do the exercises too much, when I felt like I still had the capacity to do more. So in that way I felt it was a little bit of an negative. I found one, there we go.

**Q:** Yep. No, that is good feedback. Perfect. What about would you be willing to continue with this project, or program sorry, after it’s completed?

**A:** Yes, of course. Yeah.

**Q:** Great. Would you be willing to pay for the device at your own cost? It’s normally about $150.

**A:** I probably would, now that I’ve used it. I wasn’t convinced at first because I’ve got Siri and I’ve got internet and everything. And I’ve got a Google Home. But now that I’ve used it, I probably would.

**Q:** Okay, that’s really good. Do you have any issues regarding the data privacy?

**A:** It did concern me, because you don’t want to think that you’re being listened to all the time and watched all the time, however it was alright once I found where to turn off the camera. The other thing I was a little bit concerned about was when I imported my phone or attached my phone to it, it still had all your contacts in it. So I thought that was really bad, because I didn’t want all my contacts to be in your contacts. So I didn’t like it, so I disconnected my phone from it.

**Q:** Okay. I was unaware of that. That’s really interesting, actually. Anything else?

**A:** No, I think that’s about it. To me it was quite a pleasant experience. It did get me exercising. It gave me the knowledge to have a few exercises in my back pocket ready to do any time I felt like it. So I think it was overall a good experience.

**Q:** Perfect. That’s great. We’ll stop the recording there. That’s great.

**postS008**

**Q:** It is the 15^th^ of December at 11 o’clock. We are with participant 008. The first question is how was your experience during the project?

**A:** Yeah, it went good.

**Q:** It went good. Did you find enjoyment? Did you find benefits?

**A:** I definitely found benefit because it – [it’s not so much the reminder]. I didn’t need the reminder. But I benefited because – as in – that [I’m a nervous driver]. I’ve got to [unclear] sticking to my partner if I [unclear] without holding on the rail. So it was good, yes.

**Q:** Any other positives that you can think of?

**A:** That someone was talking to me when you live on your own.

**Q:** Yeah. Did you like the –

**A:** [Unclear].

**Q:** Yeah, certainly. Did you like that you could activate through voice and not through swipe or touch?

**A:** Oh yes, yes, I didn’t need to be close to it to do the exercises. I could do it from a distance and it responded to my answers. Although, at times, it didn’t [unclear] 00:02:14. I don’t know whether it’s because – maybe it records my [unclear], which was a more – [a deep yes]. And other times when I’ve been doing the exercises and I’ve just finished, I might go, “Yep,” not the same tone, so repeated it several times. So that alone was [Unclear] got worse, where I had to just think, stop, remember I have to say yes [for the first time]. That’s about it, yeah.

**Q:** Can you think of any other positives?

**A:** [Unclear]. That it’s an activity that helps you to get along, especially during the lockdown. It was fantastic during that time. We had something to look forward to to do and it was beneficial to your health.

**Q:** Great. Did you have any negatives of using the Alexa device to deliver the exercise program?

**A:** No. But sometimes I felt [the question that was said]– when you have done the exercise and it didn’t like your yes, it said, “Do you want to continue?” It could be misunderstood – do you want to continue with your exercise or do you want to continue with the same – repeat the exercise. And obviously, it meant that it repeated the exercise. So what I learned that – when I want – when it didn’t register my first yes and said, “Do you want to continue?” and I said yes, we went back to the same exercise again. But then I learned if I said no, not wanting to continue, it just went onto the next exercise automatically.

**Q:** Yeah. No, that’s good feedback.

**A:** I think that’s a little bit misleading because they’re saying, “Do you want to continue?” [Unclear] yes in the beginning, [unclear] to the exercise that I’ve just done. And so I took the risk and thought: Stuff it, I’m going to say no and see what happens. And when I said no, it just went straight to, “Your next exercise is – [unclear].

**Q:** No, that’s good feedback. Any other negative?

**A:** No. No, that was good.

**Q:** Good. Good. Any other comments around that?

**A:** The voice of the instructor was pleasant, friendly. Yeah, no, it was nice. It was easy, easy on the ear. Generally, when you go and do exercises, then the instructions and the directions are more sharp and sometimes to the point of aggressive and pushy, whereas this one wasn’t. It was [really] gentle.

**Q:** Good. Would you be likely to continue with this exercise program?

**A:** I think I’ll do it on my own as I do [unclear] in the past. I went away on the weekend, and Sunday, I wasn’t here. But I still did all the exercises that was – [being in that program] 00:08:38. It was very easy to do [unclear].

**Q:** Good. Do you have any issues regarding the data privacy?

**A:** I’ve been hacked so many times. I’m so reluctant. But I mean the data you have on me is [readily] available everywhere anyway.

**Q:** Yeah. Absolutely.

**A:** I mean nowadays, it’s like – when we went to restaurants in [Heathcliff]– and as soon as we walked in, [unclear] welcomed us on a computer and their phones. And it’s like we’re being tracked wherever we went anyway. It’s ridiculous.

**Q:** That’s exactly right.

**A:** Yeah, especially where you have to check in wherever you go.

**Q:** That’s right. So –

**A:** Yeah. And some of the places, to my surprise, they wanted your e-mail. I didn’t know why they wanted your e-mail. I could understand name and phone number and the postcode. Yeah, which to me, it narrows it down that they can then tell exactly where you are, where you’ve come from. Yeah.

**Q:** So using the device during this project really didn’t impact your view on –

**A:** No. But what I did though, I did switch it off when I wasn’t using it, not because I have any – anything that’s going to be overheard. But I did use it several times for music background, and that was really nice, just to relax and just put it on. But I can see that it has a lot of other access to information [unclear] if I wanted.

**Q:** Did you use any of the reminders or anything like that? Any of the –

**A:** No. No. I could see that – oh, I think it might be working. Is it 11:17 now?

**Q:** It is, yeah.

**A:** Yes, it just came up. Alexa, open my [phoning buddy]. There, it’s still working. [Unclear] 00:11:13 doing more exercises. [Unclear] [doesn’t matter] which way you want to do it. Now you want to continue on the phone?

**Q:** No. Yeah. No, the phone’s great. That’s fine.

**A:** Okay. Good.

**Q:** Would you be willing to purchase the Alexa device to deliver the exercise program at your own cost?

**A:** I don't know. Given that I can do it with or without – I plan – now that we’re a little bit freer, I’ll be travelling nearly every weekend. I’m planning to do Victoria. Every weekend go somewhere. And then next year, I’ve got a few weddings in New South Wales, and I’ll be going for a few weeks, probably won’t be – I don’t really need the exercise, as such, to prompt me. I know what – I mean I’ve been doing it now, what, for nearly three months. Yeah. Yeah. And it’s fairly simple and straightforward to remember and do.

**Q:** Yeah.

**A:** Yeah. If I was to purchase it, what is the cost?

**Q:** It’s about 150.

**A:** Okay. I mean if I wasn’t planning to travel and go around, now that hopefully we can, probably yes, if I was just going to – not go anywhere or if I was to [unclear] 00:12:48 in a state where I’m not going to be moving around for at least six, seven months, yes, it will be good. But I think I won’t be using it as much as I have in the last three months.

**Q:** Yeah, makes sense. Great. Do you have any other further comments you’d like to make?

**A:** No, not really. So what happens given that it’s only over months and the program is still going? Do you disconnect it once it’s done, and then I can’t access anything anymore? That that will be the end of it?

**Q:** I’ll just stop the recording and then we can have a chat.

**A:** Oh okay, sorry.

**Q:** No, that’s all right.

**postS009**

**Q:** So the date is the 21^st^ of December at 2:00. We’re with participant 009. First question is how – what are the positives of using the Alexa device to deliver the exercise snacking program?

**A:** Well I think the advantage of having it there, knowing that it was going to remind me to do my exercises was a good thing, because I do have a physio app on my phone, but I have to actually go onto it to use it, where this actually would be reminding me. I feel very guilty if I didn’t do it.

**Q:** Any other positives that you can think of?

**A:** Well it’s good to see a demonstration – not that I needed it after the first couple, but I think for some people that’s a really good thing, yeah.

**Q:** Yeah, good. Anything else?

**A:** No, nothing else particularly.

**Q:** Good. What about the [inaudible] that it was sort of voice activated and not touching or swiping. Do you think that was a good thing or not, or?

**A:** Yeah no, it’s good. You don’t have to touch it. It just – sometimes Alexa was a little bit naughty and wouldn’t turn off and I tried to turn her off but she wouldn’t, so. But apart from that, no, it was good.

**Q:** Good. Any sort of negatives of the device?

**A:** No, not really. I mean it’s quite a little device. I think it. It’s good.

**Q:** Great. Did you ever -

**A:** It’s got a very clear screen.

**Q:** Good. Did you use it for other means like setting reminders yourself or checking the weather or something?

**A:** Yeah, and I checked the weather and I’d often play music. And the sound coming out of it is very good.

**Q:** Oh good.

**A:** The music.

**Q:** Great.

**A:** Yeah.

**Q:** And it being dispersed throughout the day, how did you find that?

**A:** Yeah, no it’s good. I think I made a mistake with the 9:30 anyway because I didn’t realise I’d made one 8:30, the next one 9:30 and the second – or third one 4:30. So the 9:30 was a bit soon after the 8:30. But anyway, I was doing more than what you asked anyway, so that didn’t matter I suppose.

**Q:** Good. Any negatives of the actual exercise snacking program?

**A:** I mean I suppose – I think I probably said last time was you don’t get feedback, so for somebody who’s not maybe that good at doing their exercises, I don’t know how you would be able to but it would be great if they would get a bit of a feedback on it, whether there’s a physical way you could get somebody to do the exercise on a certain spot and put the camera on a certain spot and then it would be able to measure – or what, I don’t know. Maybe a future version.

**Q:** Yeah, absolutely. Yeah. Anything else you can think of around that?

**A:** Well I mean people use – what do you call it, the little device with the little things on the television and play golf. I mean that’s the sort of thing that I think it’s really good, and I don’t know if you could use – if this could be developed a bit more like that, because people are actually using their muscles and getting feedback, yeah.

**Q:** Yeah. So you would like that sort of real-time feedback back to the practitioner that they could actually respond and feed back to you accordingly?

**A:** Yeah, even if it was saying “Too fast. Too fast,” because that’s what I tend to do, everything too fast. Or you know, it might say “Just one more,” you know, that sort of thing. Yeah, I think that sort of feedback would make it even more engaging for people.

**Q:** Good. But do you feel sort of a little bit stronger or fitter after the last 12 weeks?

**A:** Well I think it’s – is has been a good practice to just do it twice a day, yeah. I mean I have missed the gym, so it’s been good to have that during this time.

**Q:** Yeah, great. Do you think you’ll be likely to continue with the program?

**A:** I don’t know. I don’t know what the program would be, so I don’t know. Is there a program that continues, or?

**Q:** Yeah, well in theory you can still access the program after the 12 weeks, so do you think you’ll be likely to continue?

**A:** Oh yeah, if it continued on I probably would use it, yeah.

**Q:** Oh good.

**A:** It just gets you out of doing – yeah, it’s probably worth it.

**Q:** Yeah, good. Do you think this could potentially replicate the gym or do you feel the gym is more -

**A:** No, well I use a lot of weights in the gym, so I mean I don’t go to the gym to socialise, so I don’t need that part. But I’m not sure, because I need certain equipment, like I use the step – stair, and whether there’s exercises that would replicate a stair, I don’t know. But I guess you’d have to consider weights then and you’d need one of those elastic bandy things, which I’ve got. I’ve got those, but if you – I suppose if you clocked in for a particular program and it says, “You will need these items in order to do this level” and you would purchase them and you would have them there and you would do it. So I guess that would be the way you’d work.

**Q:** Yeah. Okay. Great. Do you have any issues regarding data privacy using the Alexa device?

**A:** I have no idea – because I had the camera turned off, I don’t know if she’s listening to me all the time. I don’t know. I don’t know if there’s privacy issues. But yeah.

**Q:** But it wasn’t a concern for you really over the last 12 weeks, do you think?

**A:** Well I had the camera turned off, so I’m not sure – and because I live alone, there’s not too many conversations going on around a machine, but if you had a private conversation you probably wouldn’t have it in front of Alexa. But I don’t know. If you haven’t turned her on I don’t know if there’s any recording happening anyway. I don’t know. I don’t know of that privacy.

**Q:** No, well yeah -

**A:** I’m always a bit suspicious of things with cameras on them, but -

**Q:** Well if it’s off then there’s nothing being recorded, so that’s good.

**A:** Yeah. It would be very interesting anyway.

**Q:** Would you be willing to purchase the Alexa device at your own cost if you were not given one?

**A:** Probably not. I probably wouldn’t buy one, no. My daughter had her nose out of joint because I got this one, and then she just bought one.

**Q:** There you go.

**A:** Yeah, so – but yeah. No. Probably not.

**Q:** Okay.

**A:** Do I have to buy it?

**Q:** No, it’s yours to keep.

**A:** Oh, okay. Good.

**Q:** No, it was just a question for future studies I guess.

**A:** Well I suppose people would need to know – if you’re going to ask them to buy, I guess they’d need to get more – there would need to be more value for the machine, like I’d need to know what else I could use it for. And yeah, I think that would be helpful. If you had a deal where you had to be part of this thing but you buy it, they need to see what other value you get from the machine and then you might get them to buy it.

**Q:** Yeah. Yeah. Do you have any other sort of comments regarding the positives or the negatives of the project?

**A:** I think it’s not a negative really, but I think – I know you added two or three more exercises. I think I probably would have liked, I don’t know, 25 exercises rotating around, because I seem to get the same ones – I mean I know there’s probably only 12 anyway, but – and then the, “Oh no, I’ve got to do that squat one again. No.” Yeah.

**Q:** So more variability?

**A:** Hmm?

**Q:** So more exercises? You would have liked that?

**A:** I think having more variety of exercises would be good.

**Q:** Yeah.

**A:** Probably one thing I would have suggested too is because I do some of these exercises with the exercise physiologist anyway, I know how you’re meant to do them. But for some people, they probably need a little bit more guidance on some of the exercises. Like I know you do say “hold your bum in” or whatever, but some of them it’s quite important that they do it correctly. So there may – but you wouldn’t want to hear that every single time. So it’s tricky because the first time they do it they need the full instruction and then after that you’re just say reminding them what the exercise is, so.

**Q:** That’s exactly right. Yeah.

**A:** Yeah.

**Q:** Any other comments around that or anything else?

**A:** No, that’s all.

**Q:** No, that’s it.

**A:** No, that’s fine.

**Q:** All right. I’ll stop -

**postS010**

**Q:** We're with participant 010, it's 22 December at 10 o'clock. The first question is what were the positives of using the Alexa device to deliver an exercise program?

**A:** Can you repeat the question, please? Yeah.

**Q:** What were the positives of delivering an exercise program using the Alexa device?

**A:** The positives, one thing, you can access the receivers, people like me who live on their own in their own houses. I mean, yeah, this is one thing. Another thing, it's a good device, no problem with connectivity and it is the first time today, I don't know why, maybe it's my internet, and the voice is clear, the subjects that I can - the questions I can ask, I mean, in addition to the exercises, I can access a lot of data, even photos and yeah.

**Q:** So outside the exercise, did you use it for other stuff like you just mentioned? So reminders, weather, that kind of stuff?

**A:** I used it - well, 98% for the exercises.

**Q:** Great. Any other positives for the exercise?

**A:** Positives in the exercise?

**Q:** Sorry, positives with the device use -

**A:** Positives of the device. It is handy, small, handy, small enough, small enough and it doesn't take much space and it works very well, it works very well. It looks like - I mean, to visitors, they think it is an alarm clock but it's not of course. Yeah.

**Q:** Good. And you liked that you can activate it through voice and not through touch, was that handy?

**A:** Yes, of course, yeah, yeah.

**Q:** Any other positives that you can think of?

**A:** Well, clarity of picture, I mean in the screen, clarity of the screen, and the quick responses that it gives when I give some instruction. The quick responses, yeah.

**Q:** Great. Did you find any negatives of the actual device?

**A:** No.

**Q:** No negatives. What about the actual exercise program itself? So the exercise snacking, the short bout frequent exercise, how did you find that?

**A:** Sorry -

**Q:** That's great.

**A:** They work better now after the exercises. Yeah.

**Q:** That's really good. Any other positives of the exercise program?

**A:** [inaudible] in the sense of - well, you took - it was mainly on the lower part of the body, on the legs and feet and ankles, there was only one exercise for the shoulders, which is wall push up. Well, which is not a negative point, it's just a [positive] point, considering the fact that people between 69 and 80 usually lose or [they didn't get] the energy of using the legs sort of diminishes and which is perhaps, well, more important than using the shoulders and the hands because no movement would be possible without the ankles, the feet, the legs. Yeah.

**Q:** Yeah, that's really good feedback. Did you find any negatives of the actual exercise program?

**A:** No, but at the beginning, maybe first month when you talk to me, there was that squat on one foot, on one leg and stand up. I think I don't know about the others, I don't think anybody can do it. I mean, I just - well, I weigh 100 kilograms, so it's not possible for me to go down and to go up. And it was the right thing to do to change that, to remove that part of the exercise and give me something else.

**Q:** Yeah. Okay, great.

**A:** So that is - I mean, the study, although you designed the exercise, you were open to make any changes according to the individuals.

**Q:** So you like that flexibility?

**A:** Yeah, flexibility, yes. But flexibility was [inaudible] maintaining the main - the core exercise [inaudible].

**Q:** That's good. That's really good. So overall, sort of no real negatives of the exercise program?

**A:** [inaudible]. I mean, I asked you the last time you talked to me maybe two weeks ago if we would be able to continue with the exercises after the study is finalised, you said - well, now [Jack] yesterday told me that - [Doctor Jack] told me that we can keep on. So I'll keep on.

**Q:** Good. So that was my next question. Would you be likely to continue with the exercise program?

**A:** Yeah.

**Q:** Good. That's really good.

**A:** Yeah.

**Q:** Would you be willing to purchase the device at your own cost?

**A:** Sorry?

**Q:** Would you be willing to purchase the device at your own cost?

**A:** You mean you want me to pay for it?

**Q:** No. You've been given the device for free but in hypothetically would you be willing to pay for it?

**A:** Yeah, it's not expensive. It's not [inaudible] it's within - yeah, not expensive, but I would be willing to buy one, yeah. I mean, and I'm sure my friends, if they know they can get access to the exercises, they would happily buy their own devices and then do the exercises at home. Yeah.

**Q:** That's good. Do you have any issues with data privacy?

**A:** No. No. No.

**Q:** Do you have any other further comments regarding the positives or the negatives of the project?

**A:** No negatives at all. I mean, I benefited much. You know, it gave me the chance to do exercises at home. I would go and walk and walk and walk and when I came back, there were additional exercises for me. And also I discover that although I would walk sometimes two hours for two hours that day, when I come to do the exercise, especially let's say the one stand up and sit down with the ankles, you know, moving your ankles, my ankles up. And the other one, the - let me - well, the lateral lunge and walking on heels, I notice that although I did the walking or I did the walking for two hours but there were muscles in the ankles that needed to be sort of treated, sort of needed to exercise to get stronger.

**Q:** Yep, yep. Good. Any other feedback before we stop?

**A:** No, I mean the exercise on multidirectional stepping was much liked by a number of people who I know and we started to do them just when we meet. Yeah.

**Q:** Great. So you do the exercises with other people, you've taught the other people -

**A:** Yes. Yes. [inaudible].

**Q:** That's really good.

**A:** Yeah.

**Q:** Perfect. Thank you. Well, I'll stop the interview now and we'll just have a quick chat.

**posts011**

**Q:** So it's 15 December at 2 o'clock. We're with Participant 011, and the first question is overall how was your experience during this project?

**A:** It's been – yeah, it's been pretty good. The last few days I've been caught up with other things, but I have been doing one or two exercises, plus the 5BX, which – let's see, where am I up to? So in the 5BX, I'm up to [A-] 00:00:45 today of the second chart, so – and I've actually passed the level that they say for I think 65-year-olds or something, up to 65, and they don't have anything for older people. But I just keep plodding on. [inaudible] 00:01:09 -

**Q:** Good. What do you believe were the positives of using the Alexa device to deliver the exercise snacking program?

**A:** Sorry, I missed part of the sentence.

**Q:** What do you believe were the positives of using the Alexa device to deliver the exercise snacking program?

**A:** Yeah. Like I said, I think - I mean it worked well, it's just that without a battery I can't - it's not portable. So I actually - those exercises I sent you that were on the web would probably work a bit better, but I've found the old book anyway, so I'm doing it out of a book, which I think is better again. Because you just start it and you don't have to - it describes how to do it anyway and towards the end of this one, because it was freezing so much, I just go through all the exercises and then wait for it to ask me do I want to continue and stuff.

**Q:** Yep. So whether -

**A:** So yes -

**Q:** Sorry?

**A:** Certainly to start off and to show what the exercises are, it's been good. It's just I think you could do that online.

**Q:** Yeah, yep. So you don't really necessarily need the device to deliver the program, do you think?

**A:** No.

**Q:** Okay. Did you use the device for reminders or checking the weather or anything like that? Any other -

**A:** Not really. I have used it for news and stuff but yeah, I've been caught up with other stuff lately, so haven't done that a lot.

**Q:** Yep. Do you like the aspect that the Alexa device is voice activated compared to swiping or touching or do you feel that doesn't make a difference to you?

**A:** Sorry, you're cutting out a bit. Like the Alexa does what?

**Q:** Do you like that the Alexa is voice activated compared to touching or swiping?

**A:** Yeah, I like that except that because of the freezes and stuff, it quite often says, "Oh, I didn't hear that. You must answer yes or no" and then it takes another 10 or 15 seconds before you've said the same thing that you just said. And I think it's to do with - it's to do with the freezing. Alexa's doing as best as it can but - and also it says, "Now, the next exercise will be blah" and it goes through the spiel and then says, "Are you ready to begin?" and you could almost have done some of the exercises by the time you begin.

**Q:** Yep, okay.

**A:** So it's certainly good at first but there should be a like shortcuts.

**Q:** Yep, okay.

**A:** And now I've - it hasn't been like that for all the time but now they just go - they alternate in the same sort of way. So like I did some - one lot of exercises today and I knew where I was up to but so let's say you start off on the four point kneeling, then it goes through the same exercises each time. So I don't actually need the Alexa anymore to know - unless the exercises change. I can just go through the four by myself. But I'll do it for another while anyway using Alexa, just because I've got into the habit of it.

**Q:** Yep, yep. Did you like the aspect that there was reminders or do you feel that didn't help that much?

**A:** I didn't hear any of the reminders. I turn it off in between sections. So it hasn't made any difference to me.

**Q:** Yep. As far as the -

**A:** And because my timing is so irregular, well actually I've been starting generally by about nine I've done my first exercise for the last week or so. Not today but most days. But the ones after that, depending on whether I go to the next town, I'm there all day, so I might be finishing the last one at 11 or just collapsing on the couch and not doing the last one.

**Q:** Yep, yep.

**A:** So because the timing is so unknown but, you know, sort of fairly regular, the reminders don't make a difference. I might've done the exercise at nine and it'll tell me that it's ready for my 10 o'clock session.

**Q:** Yep.

**A:** So, you know -

**Q:** Yep. Did you find any other negatives of the device?

**A:** No, the main negative was it may be my connectivity here, I notice on Saturdays it always goes bad for the last three or four weeks, when people have been escaping from Melbourne and bringing their devices down, swamping the bandwidth. So it's not really about the device but the main thing is the battery. So yeah.

**Q:** Yep. Okay. What about the actual exercise program, the short bout frequent exercise? Did you -

**A:** Sorry, I just have to close the front door.

**Q:** The short bout frequent exercise, did you find that of benefit? Was that a positive?

**A:** The what?

**Q:** The snacking?

**A:** Short bout -

**Q:** Frequent, yeah.

**A:** Whatever. Yeah, no, that was good. Yeah, I liked doing it like that.

**Q:** Okay, great.

**A:** I've got to a point with the 5BX one where I would have to - when I get to the next chart, it'll take more days in each level, because that's got a bit of - the amount of [bite] 00:08:53 increases quite a lot more than the snacking does. But this has been good. So like I said, I can't do the push ups very well, I can do the wall push ups, it's no problem. But and I was doing them off the couch, you know, just side of the couch here, can see it.

**Q:** Yeah, no -

**A:** Just the arm.

**Q:** Yep.

**A:** But the other one started off with half knee push ups, which was fine. And then now I've got on to proper push ups, which I used to be able to do, I don't know, 90, a hundred or something but now I can't - I tried to do 10 and there was a problem. But now that I've built up, I've built up to about 17 of those.

**Q:** That's really good.

**A:** So it's not really on the snacking -

**Q:** No, no, that's all right.

**A:** - thing but it's a similar kind of -

**Q:** Yeah, yep.

**A:** The fact that you're doing it and you're building up a bit.

**Q:** Yep, yep. Were there any negatives of the exercise snacking concept?

**A:** No, just sometimes you're caught up, you just can't do it.

**Q:** Yep.

**A:** It's not a negative, it's just life. In fact, doing it every single day is – like I've got to get my house ready for the rentals now, and I sort of – I was on the roof for about three hours the other day, and then didn't feel like doing anything much. So, you know, there's other stuff gets in the way.

**Q:** Yep. Would you be likely to continue using the device?

**A:** Yeah. Well, I mean I have been. Like I said, it ended Sunday but I've still been doing the exercises still.

**Q:** That's very good.

**A:** So I'm using the device.

**Q:** Yeah, great.

**A:** But I'd probably - if the Saturdays are so bad, I'd probably not use it at all on the Saturday just because well one time it took three hours to get through and last Saturday it took one session when I waited for it, a good three quarters of an hour. So the snacking thing doesn't work when your connectivity is not good.

**Q:** Yep, that makes sense.

**A:** But yeah, I'll keep on using it for the time being.

**Q:** Great. If you were not given the device for free, would you be willing to purchase it at your own cost?

**A:** No, I wouldn't, and especially since I've kind of - you know, I've got my old phone, my old last two phones, my present phone, an old tablet, on a really old table as well, and plus my laptop. So I would be much - I think it's a much better idea to put it onto the web, honestly.

**Q:** Yep.

**A:** And also, I mean sometimes - well, it does wait - no, it doesn't really wait for you to get ready. It just says it'll start in 10 seconds or something. So that's the same as being on the web. Once you start, you have to go through the whole session of snacks anyway. And I guess one thing you can do is hit the pause if it's on a web thing.

**Q:** Yep.

**A:** For example, when I have to do the sitting down, I drag the chair out and put it in place and stuff like that. There's plenty of time but I can just do that within a couple of seconds and maybe pause it for a couple of seconds and then keep on going.

**Q:** Yep, yep.

**A:** So what are you intending to do with it?

**Q:** Sorry? What are we intending to do?

**A:** What are you - yeah.

**Q:** Not quite sure yet, to be honest. Did you have any other comments that you'd like to make?

**A:** No, I think I commented on the various other things. It did do a few times it's in the last couple of weeks, it's started saying, "Hello, Matthew," and then stopping [inaudible] 00:14:33. Yeah. But who knows whether it's my connectivity or whether it's connectivity on the other end of the line.

**Q:** Yeah.

**A:** But it's funny that it does that but, you know, it recognises that I'm about to start a session and then just sort of drops out. And the other ones are just sort of for some reason I find it hard to - that first - when I do the bridge, to actually get my head down on the ground. There's a bit of stiffness around the shoulders but when I get time I'll go to a physio and stuff.

**Q:** Yep, yep. Did you have any comments regarding data privacy? Do you feel - were there any issues using the device and data privacy?

**A:** Well, I wouldn’t leave it on in between sessions and I wouldn't leave the video on at all.

**Q:** Yep. Perfect. Do you have any other comments - sorry -

**A:** So I guess there's times when it's been listening to a whole lot of - when I haven't turned the sound down and that, it's listened to all of my TV programs. Then again, it's a smart TV and it's recording all of that anyway, so it's just as importunate as anything else.

**Q:** Yeah, exactly right.

**A:** [inaudible] 00:16:19 especially. It's sort of really amazing when you just have a passing comment to someone during the day, your phone picks it up and then there's an ad about it two hours later.

**Q:** Yeah, exactly right.

**A:** Find that very intrusive.

**Q:** Yeah, exactly right. Do you have any other final sort of comments regarding the project?

**A:** No, I'd just be interested to see what the outcome is at some stage.

**Q:** Yeah, absolutely. Well, I'll definitely - I'll just stop the interview.

**postS012**

**Q:** We’re with participant 012 and it’s the 22nd of December at 12 o'clock. The first question is: how did you find the exercise intervention delivered by an Alexa device?

**A:** On the whole really good. There are a few hiccups. Like, there were days when Alexa said, “Sorry, there’s a problem,” and other times when Alexa didn’t really understand. I would say the set thing which is, “Alexa, open up my training buddy.” - hang on, I’ll move away from Alexa because I think she’s getting confused now. Yeah, so I’d say that and then she’d say things like, “You mean your pet buddy or your” this buddy or that buddy. Like, there’s obviously other buddies, and yeah, there was a lot of confusion and it couldn’t sort it out, so I’d have to just leave it. And that made it hard because it meant that sometimes I just didn’t get a chance to go back to it, so instead of the last four weeks doing the four sessions I might have done three or two when there was a hiccup like that. But otherwise, apart from that issue - well, those couple of issues, the rest was all right. Yeah.

**Q:** So, as far as positives, how did you find it? Was it easy to activate it through voice? Was that a good aspect of it?

**A:** Yeah, a very good aspect. Yeah, apart from the times when she didn’t understand what I was saying which was fairly rare. It might have happened on the whole over the 12-week period - I didn’t keep a diary unfortunately. I got out of hand and couldn’t find paper, couldn’t find a pen at the time and all that rubbish, but probably it might’ve happened 10 to 12 times in all, so no big deal over a 12-week period. Yeah, I thought the voice activation was really good and I thought the way the program was delivered - I know it was repetitive, showing - you showed the sequences over and over again, but I didn’t mind that. Sometimes it gave me a bit of time. Instead of doing 10 of whatever, the pogo jump, I would start beforehand, so I might do 20 which was good, so then if I missed a session, session 4 at the end, I didn’t feel so bad about it.

**Q:** Any negatives of the device or the exercise program?

**A:** Well, relating to the exercise program I think that was the only thing. And also with doing bridge, sometimes I’d have to go from where Alexa was to where the carpet was and it didn’t give me enough time to get up and down, so I’d sort of, you know, [inaudible] or I’d have to start it early or something. That was the only one re the exercise. And the device on the whole was fantastic. Just a couple of times it would say, “Sorry, there’s a problem,” and so I just had to leave it but then I didn’t get back to it. But as I said, there were two issues with it. Sometimes it couldn’t understand what I was saying [inaudible] “Alexa, open my training buddy.” 90% of the time it did; sometimes it didn’t. And the other one is when I said that, it would just obviously get confused and say, “Sorry, there’s a problem.” So, there are a few bugs, but not major as far as what my experience was with the device.

**Q:** And you touched on a little bit as far as the short bout frequent exercise, the exercise snacking. Do you have any other comments regarding that, so positive or negative?

**A:** Say that again?

**Q:** So the actual exercise snacking program, the frequent short bout exercise, do you have any other comments regarding that? You’ve spoken a little bit about it.

**A:** No, no. You mean the frequency?

**Q:** Would you be likely to continue with the exercise program after this project’s finished?

**Q:** Do you have any issues regarding data privacy?

**A:** I don’t. I know that some members of the family have said to me, “You know, they listen in on you,” and I’ve said - I’ll brush it off and go, “I don’t care,” but you could be. How secure is it from your -

**Q:** Well, from our understanding it’s - you can only really - we provide very minimal data, so not even your name is being registered anyway, so -

**A:** I hope it’s fine. I mean it does annoy me here something when I speak to it. Like, sometimes I found the device itself like my god, you think it’s fantastic but on occasions like I’ve been doing housework or something and just said, “Alexa, put on eighties music,” or whatever and their selection’s really great and the sound is fantastic and that part of it is fun. A couple of times I’ve said to it, “Give me my horoscope for today,” and that’s really good. I mean, I don’t know, does that only recognise when I speak to it or what, from a privacy point of view? You know, hearing when I’m having a conversation with someone, for example, in the other room?

**Q:** To be honest, I’m not entirely sure. I don’t believe so.

**A:** Yeah. I think other people, it could be a concern. It’s not a concern to me, but I think some people would be concerned because I have heard some people say it listens in on you and stuff like that, so I don’t know. But for me it hasn’t been a concern. I suppose maybe if it were to run again or in a different form it might be important to find out the level of privacy.

**Q:** Absolutely. Would you be willing to purchase the device outright at your own cost if you were not given one for free?

**A:** After having used it, after having a little bit - and I probably haven’t really explored the full extent, which I will do after Christmas, I would say if the price - I think you mentioned 140 - yes, I would. But I wouldn’t have purchased it because I try to limit my devices and [inaudible]. I probably wouldn’t have purchased it had I not known, you know what I mean?

**Q:** Yeah.

**A:** And also I’d be more willing to purchase it if I knew what else it could do. Like, I mean even if maybe in a less rushed situation say if you had people coming in one or two at a time and just someone that’s technically savvy said, “It can do this, this and this.” Like, I’ve never been one to read instructions, I must confess. So I’m not sure, but even just something like what’s my horoscope for tomorrow, play me eighties music or I don’t want to listen to Sky News, but ABS News or something like that. That would be really good because then you can see what the device does as well as how the exercises are displayed, how you actually showed how to do the exercise because I mean I think that was really, really good. Yeah. So, I would - yeah, I would purchase it if I had a better understanding that if I went into - say, in particular answering the question regarding the exercise - if I could see how it’s set up and see how you display the exercises and arrange and like maybe half an hour - three-quarters [in - at Deakin] - sorry, that’s the grandchildren

**Q:** That’s all right. No, that’s good. So you would like to leave the exercise program yourself. Like, would you like to have access to the exercise list and deliver -

**A:** Yeah. And just maybe drop one or two things that I’m not interested in doing, like the vertical jumps. I mean if it’s something that still could be substituted for that actually like reasonably equivalent, still exercising the same muscles or something like that or the heel walking which I’d drop and put something else in instead maybe.

**Q:** Great. Do you have any other comments, positive or negative, regarding the project?

**A:** No. No, not really. I think the level of contact with you and Jack was for me personally appropriate, fine. I was happy with it. I knew that if there was an issue like I could always ring you up. You were very reachable, I suppose is the word, so yeah. No, I don’t. I was really happy. I’d like to see maybe if I could explore other things on it like if there’s - I know there’s a lot of things like exercise programs that you can do on YouTube or stuff like that. I wouldn’t mind just exploring that myself or maybe even one of the side things that could be done through this is make some emails or send through the device some suggestions on how to click into them.

Now, that sort of leads me to another point. Like, I think this was a very general program and as a very generic program it worked pretty well, but to fine-tune it maybe a little bit better, one, because you’re working with old [farts] like me, you need to know what their individual injuries are. Like, if there’s some limits to what they can do because of some problems. Like, I think one of the things that it’s really not suitable for me to do the vertical jumps very well is because my knees. Like, I’ve had issues with my knees for the last 20 years, so I just do this tiny little hop, so that’s the reason for that. Then I slowly, slowly improve, but my left arm I had a lot of issues with it, so doing - I was attending a physio for a long time, and it was even worse. It’s like 90% better now, but it’s taken months, but I don’t know if I mentioned it. That was one of the issues.

Luckily there was nothing in the exercise program that aggravated that issue, that physical problem, so that wasn’t an issue for me apart from the knee thing, but I’m sure that if you’re in your late sixties, early seventies, whatever, you’ve usually got some problems, some part of your body that doesn’t work very well. So I think it’s important to know that beforehand.

**Q:** I’m happy to finish up there if you’d like or if there’s anything -

**A:**  Yeah.

**Q:**  - else that you’d like to add. I’ll finish up there.

**postS013**

**Q:** It’s the 4th of January, 2021. It’s 10 o'clock. We’re with participant 013. The first question is: what do you believe were the positives of using the Alexa device to deliver the exercise snacking program?

**A:** Can you say that again?

**Q:** What do you believe were the positives of using the Alexa device to deliver the exercise program?

**A:** Well, you could do it in your own home. You could set it up in different locations, pretty easy to use. You could go back and repeat it. You could see very clearly what the exercise was.

**Q:** Perfect. Any other positives that you can think of?

**A:** Hang on. It didn’t take - it wasn’t like - didn’t take too long to do with using that kind of program, pretty easy to work it out, didn’t have to pay a lot of money, so you didn’t need a personal trainer or anything like that. What else? Pretty straightforward. You could see the exercise quite well, so didn’t seem to be an issue being able to do it. Once you got the hang of working with the device it was pretty easy.

**Q:** So, on the device did you use it for anything else apart from exercise, like send reminders or checking the weather or anything like that?

**A:** Occasionally, but not very often. I’m not really into technology so much. I mean maybe it’s just that I didn’t even think of things I could use it for. I mean I know my friends who’ve used that or Google and they use it quite a lot, but I just don’t think of using it.

**Q:** No, that makes sense. Was the aspect that it was activated through voice, not through touch, was that a handy thing?

**A:** Yeah. I think it was good because I didn’t have to go over to it. I could just tell it from where I was, so yeah, that was good.

**Q:** Do you feel that it would replicate anything else? So do you feel that this device would replicate or replace even a mobile phone or a laptop when prescribing exercise or do you feel that’s not the case?

**A:** Yeah. I mean probably because I’m not used to it. I mean I’ve got a mobile phone, but well, because it was small, laptop would probably be still better because it’s a bigger screen. The phone, I guess in terms of size-wise and all that it’s probably comparable to the phone, so yeah, you could use it instead of the phone, so that way if calls come through or something, but yeah, I don’t know. I mean I don’t really use the phone for exercise either, so yeah.

**Q:** What do you believe are the negatives?

**A:** Only basically in the beginning where it was - like, the instructions weren’t - like, when it asked the questions, “Did you do this or did you do that?” and it didn’t always get - you had to keep repeating it, so I mean that’s probably something that can be fixed.

**Q:** Absolutely, yeah. Any other negatives that you can think of?

**A:** Well, maybe it had to be plugged in, so you need to be where there was a power source, so you couldn’t - you had to always have a power source, so I couldn’t just have it charged and then move it somewhere else, so I was kind of dependent on one or two locations where I had it.

**Q:** No, that makes sense, absolutely. Anything else that you can think of?

**A:** I guess it would’ve been nicer if it was a bit bigger, the screen. Apart from that, no, I think it was okay.

**Q:** Great. So as far as the exercise program itself, so the short bout frequent exercise, how did you find that?

**A:** Yeah, that was okay. I mean it tended - because of the - sometimes not getting the answers you need to keep repeating and things like that, it took a bit longer than it could have, but apart from that it was okay; it was good. [Inaudible]

**Q:** So in that aspect you would prefer -

**A:** [Overtalk] then I was more likely -and then I was likely to do it.

**Q:** So in that aspect you would have preferred the sessions to be longer, those sessions to be longer but less frequent; is that correct?

**A:** Yeah. Yeah.

**Q:** Any other negatives that you can think of as far as the exercise?

**A:** As far as the exercise, no. Not really.

**Q:** Did you find it was hard enough, easy enough to [overtalk]?

**A:** Some were easier than others. It could’ve been a little bit harder as time went on. After having more sessions, just make it a little bit - you know, a little bit harder.

**Q:** Would you be likely to continue doing the program after it’s completed?

**A:** Yeah, I could. Yeah.

**Q:** Would you be likely to purchase the device at your own cost if you were not given one for free?

**A:** Probably not because you do get things on YouTube and do other things. I mean if I was more committed to exercise and technology I might, but not really that way.

**Q:** Do you feel that this would not replicate, say, having a person conducting the exercise in person?

**A:** I mean these days you don’t necessarily need to have an actual person, live person, doing it, so yeah, it could be okay, but I mean in terms of me actually going out and getting a device and doing it I wouldn’t, but it doesn’t mean that if I had it I wouldn’t use it.

**Q:** That makes sense, yeah. Do you have any issues regarding data privacy?

**A:** Well, I’m a little bit - I mean in terms of the exercise and stuff not really, but if I had to - like, if it was a different kind of program where it’s involving a lot of private things like medical stuff or opinions about things or whatever I might, yeah, think twice about that because the stuff that it’s Alexa or a Google Home thing where they do - you don’t know where your information is and all that sort of thing. Yeah, I mean I’m a bit not suspicious but I’m wary of the devices just generally, but in terms of just data for the exercise it’s not an issue for me. It’s only if it’s asking personal information.

**Q:** Do you have any other comments regarding the positives or negatives of the project?

**A:** It’s a good project to do because - especially these days with a lot of people having to be home or more at home. Something like this is probably a good thing. It’s a good thing to develop.

**Q:** Right, thank you. I’ll stop the interview now.

**A:** Okay.

**postS014**

**Q:** It’s the 22^nd^ of December at 4:00. We’re with participant 014. First question is what do you believe are the positives of the Alexa delivering the exercise intervention?

**A:** Positives?

**Q:** Yeah.

**A:** Look, I can see that it’s – get to large numbers of people, without one on one. So that’s its main – well it’s obviously the main objective, but it’s also pretty positive. I don’t really have an issue with it at all as far as the delivery side of things.

**Q:** Good. Did you like the aspect that it was voice activated or touch or swipe?

**A:** Yeah, all of those, so that if you – yeah, any of those, they suit me, because I particularly like the voice thing if you can just say it without having to go and sort of manually touch the screen, yeah. That’s good, yeah.

**Q:** Do you feel a bit -

**A:** Oh my only comment I made is that it’s – a lot of the times though I go to answer “Do you want to repeat the exercise?” and I say no and it doesn’t detect that I’ve said no. Even I’ve deliberately left a little gap there to allow to listen, but it still gets it wrong. Like it says it didn’t understand what you said, or you know? [Expects - ] or just hangs there [unclear]. It’s just happened a number of times. I thought it might have learnt my voice or something a bit better. I didn’t get the impression it was learning anything that I was saying, if you know what I mean? I don’t know if it’s supposed to learn the user’s voice or something, or?

**Q:** From my understanding, it was.

**A:** Yeah, so I didn’t get that impression, but that’s – it seemed to – it didn’t – the answer expected was yes, no, or to suspend or something or other, the expected response was you know, that’s what you get. But it happened quite a few times, but it’s okay, but it just – it usually went back to repeating the thing anyway.

**Q:** Any other sort of negatives as far as -

**A:** My only other comment would be – it’s a negative thing. I found that I would have rather hopped straight into the exercise and go forward of maybe the demo bit and the countdown and “are you ready?” and all that sort of stuff. Maybe you just hop straight – I think it might have needed an option that you could go straight into the things after a while, once you learnt it. I think that got a little bit monotonous after you’ve – so many weeks of the same same.

**Q:** Absolutely, yep.

**A:** Yeah, so it just – maybe there could be an option that you could just forget all that preamble and just get on with the exercises.

**Q:** Yeah, no that’s good feedback.

**A:** If it was satisfied – if you or someone was satisfied that you’d learnt it, you know? I don’t mean just from day one, but I mean just after – it was 12 weeks, so maybe, you know, after half the thing or [unclear]. Maybe if they had two weeks of that sort of thing and then it dropped that.

**Q:** Yeah. No, that is good feedback. Anything else as far as that or any other sort of comments around that?

**A:** Oh no. Other than that, it’s – I found it quite good. There’s a number of exercises that have dropped off that I noticed, or seemed to have dropped off, since you’ve put some more on. Like the [single knee,] handstand – not a handstand – single squat, leg squat and all that sort of stuff. There’s a few things that seem to have never come back again. I didn’t – I don’t know if that was intended or not.

**Q:** Did you use the Alexa device for setting reminders or checking weather, any other sort of additional stuff?

**A:** No, not really, because I’ve got a similar thing myself, so I’ve got – I have Alexa anyway, but I don’t use [unclear] that much. I use Siri a bit too, so Apple’s thing. Yeah so I’m not – when I set the time, I usually set – I might have set it a couple of times, but just because it’s down in the kitchen, I can – it’s handy to set her as a timer for the cooking thing.

**Q:** Yeah. Yeah.

**A:** But prior to that, sometimes my just default thing is to go to my phone and sort of [unclear] but anyway. Sometimes you forget.

**Q:** Yeah. Any other positives with the Alexa device delivering the program?

**A:** No, I thought it was pretty good. The screen, the quality’s good. The screen sounds good. I think I’ve told you before, your sound was really good. It was clear and – it was clearer than the actual – the start-up stuff, I thought. And I did take it away and it worked, so that was all right.

**Q:** So it’s pretty transportable?

**A:** Yeah. Yeah. I went away to Byron Bay and I didn’t – I only took carry on. I squashed it in there, so it was all right.

**Q:** So as far as the exercise snacking program, how do you find that? Did you find that good?

**A:** It’s pretty good actually overall. Yeah, I didn’t know what to expect, but I think it was – some are really easy. Some are a little bit more challenging, but they’re good. I think I got something out of it physically, yeah, so. The balance one is one I was concentrating on. When you do the head turn things, that really throws me off a bit, so it was getting better.

**Q:** What about the frequency, so the sort of short bout frequent exercise? How did you go with that?

**A:** Yeah that’s good, but as I say, it could even be better if you didn’t have to have all that preamble. A lot of that stuff seems to – to me it seemed to – it could even be quicker if you didn’t have a lot of that demo and countdowns and things, if it went straight into it. After a while when you got used to it, of course, I think that would have been a bit better.

**Q:** Yeah. So you felt it did not get in the way of your day-to-day stuff?

**A:** No, I did tell you that I was – the exercise it suggested it was going to come up, I got into that as the preamble was going on. So I then found myself twiddling my thumbs a bit, doing a few extra ones or something. But you know, and the question bit I – did you finish it? Were you able to complete it? All that sort of thing. Maybe it’s just that you just know or something that just – you know, at the end of the exercise rather than a lot of – too many questions. And just get on with it. [unclear]

**Q:** Yeah.

**A:** But only if you’re copping it and things. [unclear].

**Q:** Yeah. Did you – any negatives of the short bout frequent exercise?

**A:** No.

**Q:** Yeah. Yeah.

**A:** Yeah, it’s just everything’s bunching up now.

**Q:** Yeah, absolutely. Do you have – would you be likely to continue with the exercise program once it’s finished?

**A:** Yeah, I think I will. Yeah, I’ve got – I’ve written out all the exercises on a sheet, so if it was lost or something I’ve still got something to go back to.

**Q:** Yeah. Good. Would you be likely to pay for the device at your own cost if you were not given one for free?

**A:** Yeah, if I could. They’re not that dear and they’re probably coming down in price anyway, so yeah, that’s all right. But probably I was thinking more maybe if there was something similar that was an App on the phone that would probably do the same.

**Q:** Yeah, okay. So the act of using the device is not – it doesn’t replicate the phone. Is that – do you think?

**A:** The advantage that it offered is that it’s statically sitting there and gives you that reminder to do the things at those times that you’ve said. That’s a good trigger.

**Q:** Yeah.

**A:** A phone would probably do something similar by alarm, but I mean it’s still you have to then go and dive into the app and all that sort of stuff, I guess. So it’s not [unclear] but it’s similar screen size and similar – if it was on the side it would probably be the same.

**Q:** Yeah. Yeah. Do you have any issues regarding the data privacy?

**A:** No, not really, because as I – I think I told you before that I had the – I like that idea the camera can – you can cover. So that if there was any – I had that covered ever since the first interview. In fact I had it still covered when I was trying to answer Jack and I didn’t realise until I – because it showed that the camera was off, so I couldn’t – when I was trying to answer you I could see that I couldn’t see myself, so – and the camera was crossed out. So I realised that oh yes, I have to turn that camera on. So I thought that was pretty good with that there is no issue with me and privacy because of the fact you can turn the camera off.

**Q:** Yeah, that’s good. Good.

**A:** Yeah.

**Q:** Do you have any sort of final comments on the positives or negatives of the project?

**A:** Overall I think it’s a good idea. I’ve spoken to a few people and they’ve sort of said, “Oh yeah” – they’ve looked at – like they’re interested to know about snack exercising. And yeah, people are pretty – you know, I thought, “Yeah, that’s good.”

**Q:** Good. Perfect. Well I’ll stop the recording there. Thank you again. I’ll just stop it.

**posts015**

**Q:** Here with participant 015. It’s the 22^nd^ of December at nine o’clock. The first question is: what were the positives of having an exercise program delivered by Alexa?

**A:** Convenience definitely. Not having to go out. As you know, I walk regularly, but that’s rather weather-dependent, and not having to dress up in a raincoat or an umbrella or something, and also, building other muscles that I haven’t worked on before is probably a good thing. So yes, that convenience and immediacy and regularity.

**Q:** Yeah. Any other positives.

**A:** I think there must be a way to do it that you zone your head out somehow.

**Q:** Yeah. Any other positives.

**A:** Any other positives, none that I can think of at the moment.

**Q:** That’s all right. Did you use the device for anything else like setting reminders, checking the weather?

**A:** All the time. At the moment I’ve just had a cataract done and I have to take eye drops five minutes apart and just remind me in five minutes is quite valuable. If I go and do something else and forget.

**Q:** Good. Prior to having the device, how would you have done those sorts of tasks?

**A:** The microwave timer. And because Alexa says something specific, so I’ve got, “Take drops.” I can easily zone out for that timer because I use it so often and I just don’t notice regular things.

**Q:** Yeah. Do you like the aspect that it’s voice activated, not touch? Do you find that helpful?

**A:** Yes, that’s convenient. Especially now that I’ve had the cataract operated on, it’s quite hard for me to read small print so I’ve got to find glasses which are never far away.

**Q:** Nice. Were there any negatives of the Alexa device?

**A:** I think I’ve reported from time to time that are most frustrating - one of the most frustrating was the tendency for it – you very kindly set reminders for me on it which was good but quite often I would pre-empt and think, oh, it’s nearly seven o’clock, I’ll start doing the exercise. And as soon as the reminder comes up, it closes down the exercise button. If there’s some way of reminders that are exercised by the overriding reminders in a temporary capacity.

**Q:** Okay, yeah, that’s good feedback. Any other negatives?

**A:** The other thing was that I tend to answer the questions faster than Alexa hears and particularly it then doesn’t hear me and repeats the exercise. “Would you like to continue? Would you like to repeat?” And I don’t really want to repeat. That option to repeat, it needs to be responsive if someone just answers in a natural manner.

**Q:** Yeah, that is good feedback. Anything else?

**A:** No, it’s always good to have a real human being to deal with. But as a substitute, I think it’s quite good. The other thing was I did get bored. You brought in some more exercises to give some variety and I suggested explaining why we were doing these. I’m guessing that the balance is good for someone my age because falls can be a problem as you get overzealous, sort of thing. But the explanation of the value of what we were doing would be good, the physical value.

**Q:** Yeah, that’s good. As far as the exercise snacking program, the short, bout, frequent exercise, how did you find that?

**A:** I think that’s good. It’s good when you’re busy. It would be good if every minute is good, if you can shorten it, that would be good. Shorten it, to get it down to five – the thing was it often took an extra minute or so because I did the wrong answer at the wrong time or too soon.

**Q:** Any other positives. Were you able to fit it in to your day-to-day routine?

**A:** I [certainly wasn’t wrong when it was only – what was it, twice verses or something, once verse, was it?] 00:05:55 The four times a day just leading up to Christmas became quite tricky, and I have – we went to Daylesford, six of us in a car with a dog last Saturday or Sunday and I missed – what have I done with the sheet, I missed two exercises then. But I just gone back at nine at night and I did the last one. But I wasn’t going to catch up and do another two.

**Q:** Yeah. Do you reckon four was just too much. Do you reckon between that two and three is probably ideal do you feel?

**A:** Yeah, that’s leading the witness but you’re probably right.

**Q:** Okay. Any negatives.

**A:** And is there additional value in the four, that would be – is there a physical value?

**Q:** Yeah, that makes sense. Would you be likely to continue with the program after we’ve completed – after you’ve completed it?

**A:** Yes, I was just thinking you might ask that. This morning the reminder came up and I thought, will I do one before Paul rings? And I thought, no, I’ll get the washing done. I probably will but it won’t be four times a day. I will do some sometimes. Can you tell, can you do a longitudinal study in six months as to whether I’ve been doing any, I mean for participants?

**Q:** It’s not within scope, you’re more than welcome to report back and that would be really good information.

**A:** No, I just wondered if you could see remotely how many I did?

**Q:** We can actually, yeah, that’s a really good point. I will make an effort to do that actually. Do you have any issues regarding data privacy using the device?

**A:** I have turned off the camera except talking to you now. Yes, I suppose with the camera on I would have issues with that, in general, not from Deakin University, but just from Amazon up there in the Cloud, and who else is on it and so on.

**Q:** Would you be willing to purchase it at your own cost if you were not given one for free?

**A:** You did ask that last time. I don’t feel it’s – and I enjoy the music too I must say. I won’t say the name now, but face and jazz or something like that. I do enjoy that because I’m on my own. And my grandchildren enjoy asking it to play pop. Yes, I’ll have to think on that, I’m not quite sure whether I want to spend $200 or whatever for access to music rather than putting on – I still put on CDs, I’m old-fashioned. It is a convenience.

**Q:** Yeah. Do you have any other comments regarding the positives or the negatives of the project?

**A:** No, I think the concept is sound and I do hope that they go ahead with it and give them some encouragement and just keep improving it a bit.

**Q:** Yeah, absolutely.

**A:** I wondered too, when you’ve paid for exercise courses, you interact with a personality and it might be you’re doing belly dancing or something, just something that’s got a little bit more creativity in it and fun. How much did you enjoy when you say five out of five, I’d say, “Five out of five,” because I did it? But in terms of enjoyment, I’m not so sure really.

**Q:** Yeah, that makes sense. Anything else.

**A:** Not that I can think of.

**Q:** No. I’ll stop the recording.
